# Multivariate multiple regression models of poly(ethylene-terephthalate) film degradation under outdoor and multi-stressor accelerated weathering exposures

**DOI:** 10.1371/journal.pone.0209016

**Published:** 2018-12-20

**Authors:** Devin A. Gordon, Wei-Heng Huang, David M. Burns, Roger H. French, Laura S. Bruckman

**Affiliations:** 1 Department of Macromolecular Science and Engineering, Case Western Reserve University, Cleveland, Ohio, United States of America; 2 SDLE Research Center, Department of Materials Science and Engineering, Case Western Reserve University, Cleveland, Ohio, United States of America; 3 3M Company, Maplewood, Minnesota, United States of America; University of New England, AUSTRALIA

## Abstract

Developing materials for use in photovoltaic (PV) systems requires knowledge of their performance over the warranted lifetime of the PV system. Poly(ethylene-terephthalate) (PET) is a critical component of PV module backsheets due to its dielectric properties and low cost. However, PET is susceptible to environmental stressors and degrades over time. Changes in the physical properties of nine PET grades were modeled after outdoor and accelerated weathering exposures to characterize the degradation process of PET and assess the influence of stabilizing additives and weathering factors. Multivariate multiple regression (MMR) models were developed to quantify changes in color, gloss, and haze of the materials. Natural splines were used to capture the non-linear relationship between predictors and responses. Model performance was evaluated via adjusted-*R*^2^ and root mean squared error values from leave-one-out cross validation analysis. All models described over 85% of the variation in the data with low relative error. Model coefficients were used to assess the influence of weathering stressors and material additives on the property changes of films. Photodose was found to be the primary degradation stressor and moisture was found to increase the degradation rate of PET. Direct moisture contact was found to impose more stress on the material than airbone moisture (humidity). Increasing the concentration of TiO_2_ was found to generally decrease the degradation rate of PET and mitigate hydrolytic degradation. MMR models were compared to physics-based models and agreement was found between the two modeling approaches. Cross-correlation of accelerated exposures to outdoor exposures was achieved via determination of cross-correlation scale factors. Cross-correlation revealed that direct moisture contact is a key factor for reliable accelerated weathering testing and provided a quantitative method to determine when accelerated exposure results can be made more aggressive to better approximate outdoor exposure conditions.

## Introduction

Poly(ethylene-terephthalate) (PET) has garnered considerable attention in the photovoltaic (PV) and display technology industries due to its combination of material properties. The high dielectric breakdown strength of PET film is responsible for its extensive use as an electrical barrier in PV backsheets. PET as an engineering material is susceptible to degradation resulting from environmental stressors including heat, light, and moisture [1–4]. Over substantial periods of in-service or laboratory exposure, significant cracking and delamination of PET based backsheet films occurs resulting in the decline of dielectric breakdown strength and subsequent electrical safety hazards such as loss of wet insulation resistance [5, 6]. Developing data-driven methods to effectively characterize the degradation of the PET over time is desirable given the interest in implementing the material across industries. Additionally, previous research by Gok et al. has demonstrated that the combination of thermal, photolytic, and hydrolytic stressors further exacerbates the rate and extent of PET degradation [7].

Extended exposure to UV radiation and moisture drive wide-spread cleavage of ester bonds in the polymer through photolytic and hydrolytic degradation mechanisms resulting in discoloration, gloss loss, hazing, decreased molecular weight, and eventual deterioration of the physical integrity of the film [8–13]. Photolysis of PET primarily occurs through the absorption of high energy, short wavelength, UV light, which leads to chain scission of chemical bonds along the polymer backbone. Ester carbonyl groups and aromatic phenyl rings are known to be the chemical species responsible for photolytically driven events in the PET structure [14, 15]. These chemical groups participate in Norrish type I and Norrish type II reactions under photolytic conditions [1–4, 16, 17]. Stabilizers are often added to the material formulation to mitigate photolytic degradation [7]. UV stabilizers are included to slow the rate of photolysis through the absorption of UV light and conversion of the energy. TiO_2_ also aids in the reduction of photolysis by scattering damaging radiation away from the material. Hydrolysis of PET primarily occurs through the water-driven cleavage of the ester bond. Each chain scission consumes one water molecule and yields a carboxyl end group and a hydroxyl end group on the resulting PET chains. Much work has been done to characterize the hydrolysis process and develop kinetic models, for example Eq 1 which describes the hydrolytic degradation kinetics of select condensation polymers under humidity aging conditions [4, 18–20].

$$\begin{array}{c} ln\left(t_{fail}\right)=\frac{E_{a}}{RT}-ln\left(A\right)-ln({\left[RH\right]}^{2}), \end{array}$$ (1)

Eq 1. Kinetic equation for hydrolytic degradation of select condensation polymers under humidity aging conditions, reproduced from Pickett and Coyle [20]. *t*_*fail*_ is the time to failure, *E*_*a*_ is the activation energy, *R* is the gas constant, *T* is temperature in kelvin, *A* is a factor that accounts for entropy and various constants, and *RH* is relative humidity.

Methods to stabilize against hydrolytic degradation include capping of moisture sensitive end groups and the addition of moisture scavenging additives. Capping prevents hydrolysis reactions at polymer chain ends and moisture scavengers reduce the amount of water in the system. Quantifying the lifetime performance of PET is critical to the further commercialization of PET-based engineering components.

Service life prediction (SLP) involves estimation of functional lifetime for materials and system components from time-limited end use datasets or data produced from accelerated simulations of end use conditions. Service life is formally defined as the “period of time after installation during which essential properties meet or exceed minimum acceptable values” [21]. For solar materials, photovoltaic modules and power plants, these mininum acceptable values have not been rigorously defined, since PV systems typically are used for many years beyond their product warranty period. SLP involves mathematical modeling of the degradation of properties (responses) as a function of weathering stressors and time. Hong et al. discuss that there are two approaches to modeling the effects of covariates (i.e. predictors) on responses [22]. A modeling approach based on physical, chemical, and engineering functional forms can be used when there is sufficient information about the underlying functional form governing the relationship between stressors and the dominant degradation mechanism [22]. For example, if the relationship involves steady growth or decay then an exponential form could be used to model the degradation process over lifetime. Data-driven, statistical modeling methods can also be applied when there is insufficient information about the underlying functional form from scientific knowledge about a physical or chemical mechanism that dominates the degradation process determining lifetime performance. The majority of SLP models developed for polymers and solar materials have been based on known physics-derived equations, e.g. the Arrhenius equation [23–25].

Models based on the Arrhenius equation, that describes the kinetics of chemical reactions and diffusion processes, are popular in the fields of materials weathering and SLP because it is assumed that the kinetics of the underlying chemical reactions that cause material degradation also govern the change in observed properties. Pickett presents Arrhenius-type models for the photolytic and hydrolytic degradation of polymers [20, 26]. While physics-based models are favored for reliable extrapolation beyond the range of a materials actual exposure, data obtained from experiments is not always strongly described by a known physical relationship [25]. Data-driven statistical models, fit with equations not constrained by physics, have been developed for SLP of polymers and solar materials that avoid this shortcoming [22, 27, 28]. These statistical models are more flexible and often provide a more accurate description of the dataset or phenomena of interest.

Statistical models are useful to quantify the relationship between predictors and responses [29, 30]. Such models are used to characterize the effects of environmental stressors (predictors, e.g. photodose, moisture, and temperature) on material properties (responses, e.g. color, gloss, and haze) in weathering studies. Knowledge of how weathering stressors impact material properties, and the rates of impact, allows materials scientists to understand how a material’s formulation affects its functional properties and allows estimation of long term behavior. In general, univariate multiple regression modeling is one of the most common and effective methods to determine these relationships [31]. The flexibility of these models allow them to more accurately describe non-ideal data for SLP and lifetime performance prediction. The univariate multiple regression model is given by Eq 2:

$$\begin{array}{c} y=X\beta +\varepsilon , \end{array}$$ (2)

where ***y*** is an *n* × 1 column vector of observations on a response variable, ***X*** is an *n* × (*p* + 1) matrix of observations on *p* predictor variables (i.e. photodose, moisture, and temperature), ***β*** is the (*p* + 1)×1 vector of regression coefficients and *ε* is the *n* × 1 vector of error terms. Multivariate multiple regression (MMR) models build upon the foundation of univariate multiple regression model by allowing more than one response variable to be described in terms of the same set of predictors [32, 33]. In the MMR model, the response vector ***y*** is replaced by an *n* × *m* matrix of responses ***Y***. Generally, a MMR model can be represented in Eq 3 as follows:

$$\begin{array}{c} Y=XB+E, \end{array}$$ (3)

where *n* is the number of observations, *m* is the number of response variables, *p* is the number of predictor variables, ***Y*** is an *n* × *m* matrix of responses, ***B*** is a (*p* + 1) × *m* matrix of regression coefficients and ***E*** is an *n* × *m* matrix of error terms. Each column of ***Y*** represents a distinct response variable (i.e. responses: color, gloss, and haze). MMR models were used in this study to simultaneously capture the discoloration, gloss loss, and haze formation degradation phenomena of PET in one model. These models aid in the comparison of the effects of stressors and material composition across the different response variables because the predictors are consistent within a given model. MMR models were also used because they balance the two necessities of goodness of fit and model interpretability, which is further discussed in Appendix F in S1 File.

This work provides descriptive data-driven models of the service lifetime of nine grades PET films with different levels of stabilization under outdoor and accelerated exposure conditions. MMR models are compared to physics-based models, and the cross-correlation of accelerated exposures to outdoor exposures is discussed. Though these undesirable effects of exposure can be mitigated by the inclusion of appropriate stabilizing additives, degradation of PET photovoltaic backsheets remains a concern worthy of further inquiry via comprehensive characterization in conjunction with an understanding of the underlying degradation mechanisms activated under different environmental stressors.

## Materials and methods

### Materials

Nine grades of PET film were examined in this study. The films can be divided into two groups: clear and TiO_2_-filled (white) films. The three clear grades of PET are denoted as C1, C2, and C3 (Table 1). Both C1 and C2 are unstabilized grades of film. The C3 grade of film contains Tinuvin 360 UV stabilizer. The six white grades of PET are denoted as W1, W2, W3, W4, W5, and W6 (Table 2). The W6 grade of film contains Tinuvin 1577 UV stabilizer. The W2 and W5 grades of film were hydrolytically stabilized with cyclohexanedimethanol (CHDM). Films were prepared by cutting large sheets into smaller, individual samples (replicates). Films were obtained from commercial sources.

**Table 1:** Properties of clear grades of PET film. “Unstabilized” indicates that the samples are free of chemical stabilizers. “M” represents the material grade indicator variable encoding that will be used for modeling of clear films. “#Replicates” represents the number of individual replicates for each grade in the study.

Type	Stabilizer [mol%]	Thickness [*μm*]	M	#Replicates
C1	Unstabilized	254		58
C2	Unstabilized	127	M1	48
C3	0.20% Tinuvin 360	127	M2	58

**Table 2:** Properties of white grades of PET film. “Unstabilized” indicates that the samples are free of chemical stabilizers. PVC is the pigment volume concentration of TiO_2_. “M” represents the material grade indicator variable encoding that will be used for modeling of white films. “#Replicates” represents the number of individual replicates for each grade in the study.

Type	Stabilizer [mol%]	Thickness [*μm*]	PVC [%]	M	#Replicates
W1	Unstabilized	127.0	0.2		58
W2	0.50% CDHM	149.9	1.6	M3	48
W3	Unstabilized	124.5	3.6	M4	58
W4	Unstabilized	50.8	3.9	M5	58
W5	0.50% CDHM	76.2	4.8	M6	48
W6	2.30% Tinuvin 1577	50.8	6.4	M7	58

### Study protocol: Structure and exposures

A randomized, longitudinal study design was used to study the degradation of PET films. Two replicates, or identical film samples cut from a large sheet, of each grade of PET film were assigned to nine types of accelerated exposures. Fifteen replicates of each grade of PET film were divided across two types of outdoor exposures in two locations. Five additional replicates of C1, C3, W2, W3, W5, and W6 grade films were assigned to the outdoor exposures in two locations. The study was conducted on 492 samples in total.

Outdoor exposures were conducted in four month intervals at two Atlas exposure sites with the conditions summarized in Table 3 [34]. These outdoor sites are in two distinct Köppen-Geiger Climate Zones; New River, AZ is Am (Arid, Steppe, Hot) while Homestead, FL is BSh (Tropical, Monsoon) as determined using the kgc R package [35]. The two outdoor exposures types included exposure at a fixed angle equal to the latitude of the exposure location and exposure on a 2-axis solar tracker. Samples were mounted in four different configurations: open to exposure on all sides of the sample (Open), under a borosilicate float glass cover-sheet (Cover), atop a sheet of borosilicate float glass (Glass), and adhered directly to the aluminum structure of the 2-axis solar tracker (Al). The transmission spectrum of the borosilicate glass used in this study is shown in Appendix A S1 File.

**Table 3:** Summary of outdoor exposures. Fixed angle exposures are followed by the tilt angle of the exposures. “2-Axis” refers to exposures conducted on 2-axis solar trackers. “#Samples” is the total number of samples under each exposure configuration. Köppen-Geiger climate zone categories are given in parenthesis beside the locations [35].

**New River, AZ (BSh)—Low Moisture**		
Exposure	Mount Configuration	#Samples
Fixed (34°)	Open	45
2-Axis	Open	45
2-Axis	Cover	45
2-Axis	Glass	10
2-Axis	Al	20
**Homestead, FL (Am)—High Moisture**		
Exposure	Mount Configuration	#Samples
Fixed (25°)	Open	45
2-Axis	Open	45
2-Axis	Cover	45
2-Axis	Glass	10
2-Axis	Al	20

Five replicates of each grade of PET film were assigned to fixed angle exposures, the “Open” configuration variants of 2-axis tracking exposures, and the “Cover” configuration variants of 2-axis tracking exposures each. Five additional replicates of the C1 and C3 grades of PET were assigned to the “Glass” variants of 2-axis tracking exposure. Five additional replicates of the W2, W3, W5, and W6 grades of PET were assigned to the “Al” variants of 2-axis tracking exposure.

Artificial accelerated weathering was conducted in accordance with ASTM standard practices G151, G154, and G155 [36–38]. Three UVA-340 fluorescent ultraviolet lamp exposure conditions, outlined in Table 4, were run. Varying the duration of the dark, condensation segment varied the water stress in a controlled manner. A series of full spectrum (xenon-arc) exposures, outlined in Table 5, were also conducted at a range of irradiance levels and temperatures. The full spectrum exposures were conducted in an Atlas Ci5000 xenon arc Weather-ometer™ equipped with second generation daylight filters conforming to the requirements of ASTM D7869 Annex A.1 [39]. A water stress was introduced in exposure condition Xe3 by spraying the front face of the test specimens with water during a dark segment.

**Table 4:** Summary of fluorescent ultraviolet lamp exposure conditions. BPT is the black panel temperature. Designations are classifiers used in modeling and cross-correlation. “#Samples” is the total number of samples under each exposure condition.

**Exposure**	**Designation**	**Segment 1—Light**			**#Samples**
		*UVA*_<360_ [W/*m*^2^]	BPT [°C]	Duration [hr]	
FUV1	Dry-UV	61	70	24	18
FUV2	Wet-UV	61	70	8	18
FUV3	Wet-UV	61	70	4	18
**Exposure**	**Designation**	**Segment 2—Dark + Condensation**			**#Samples**
		*UVA*_<360_ [W/*m*^2^]	BPT [°C]	Duration [hr]	
FUV1	Dry-UV	N/A	N/A	N/A	18
FUV2	Wet-UV	0	50	4	18
FUV3	Wet-UV	0	50	4	18

**Table 5:** Summary of the xenon-arc lamp exposure conditions. BPT is the black panel temperature. ChT is the chamber temperature. Xe3 is the only xenon-arc exposure that included a dark segment with water spray. Designations are classifiers used in modeling and cross-correlation. “#Samples” is the total number of samples under each exposure condition.

**Exposure**	**Designation**	**Segment 1—Light**					**#Samples**
		*UVA*_<360_ [W/*m*^2^]	BPT [°C]	ChT [°C]	RH [%]	Duration [hr]	
Xe1	Dry-FS47	26	70	47	50	24	18
Xe2	Dry-FS47	60	70	47	30	24	18
Xe3	Wet-FS	60	70	47	30	2	18
Xe4	Dry-FS70	60	95	70	20	24	18
Xe5	Dry-FS80	46	110	80	20	24	18
Xe6	Dry-FS47	100	90	47	30	24	18
**Exposure**	**Designation**	**Segment 2—Dark + Water Spray**					**#Samples**
		*UVA*_<360_ [W/*m*^2^]	BPT [°C]	ChT [°C]	RH [%]	Duration [hr]	
Xe3	Wet-FS	0	N/A	47	>90	1	18

The ASTM G177 spectrum serves as a standard guide for comparing manufactured light sources used for artificial weathering to natural sunlight [40]. For ease of comparison between exposures, photodose values were scaled to the same total ultraviolet irradiance as the ASTM G177 spectrum summed over all wavelengths 360 nm and below (*UVA*_<360_; MJ/*m*^2^).

This metric (*UVA*_<360_) will be used to normalize for the differences in the irradiance distributions between the sources and allow a coherent analysis of the weathering experiments.

### Evaluations

Samples were evaluated using a repeated-measures design with property measurements made after each exposure interval. Samples were evaluated every four months for outdoor exposures and at variable exposure intervals for accelerated exposures. One replicate of each grade of PET remained unexposed and was re-evaluated periodically to assess measurement consistency. Two sample replicates were removed from the outdoor exposures at each time step, for potential additional evaluations.

Color was evaluated using a HunterLAB Ultrascan PRO d/8 spectrophotometer operated in accordance with ASTM E1348 [41]. Colorimetric values were reported in terms of CIELAB (L*, a*, b*) for CIE Illuminant D65 and the CIE 10-degree Standard Observer.

Change in color as a function of exposure was evaluated in terms of CIELAB color difference ($\Delta E_{ab}^{*}$; commonly referred to here as ΔE) and was calculated in accordance with ASTM D2244 using Eq 4 [42],

$$\begin{array}{c} \begin{array}{c} \Delta E=\sqrt{{\left(L_{f}-L_{i}\right)}^{2}+{\left(a_{f}-a_{i}\right)}^{2}+{\left(b_{f}-b_{i}\right)}^{2}}\;, \end{array} \end{array}$$ (4)

where *L*_*f*_, *a*_*f*_, and *b*_*f*_ designate the color values of a sample after a given exposure interval and *L*_*i*_, *a*_*i*_, and *b*_*i*_ designate the average color values of the unexposed sample of the given grade of PET. Polymer discoloration is commonly observed through changes in the CIELab values.

Specular gloss was measured at 60° geometry in accordance with ASTM D523 using a BYK Gardner 4446 Micro-Tri-Gloss Meter [43]. Change in gloss at 60° (Δ*Gloss*_60_) was calculated via Eq 5,

$$\begin{array}{c} \begin{array}{c} \Delta Gloss_{60}=Gloss_{60f}-Gloss_{60i}\;, \end{array} \end{array}$$ (5)

where *Gloss*_60*f*_ designates the value of gloss at 60° for a sample after a given exposure interval and *Gloss*_60*i*_ designates the value of gloss at 60° for the unexposed sample of the given grade of PET. Gloss is a generally accepted measure of surface light scattering due to surface roughness.

Haze (%) is the ratio of diffuse transmittance to total transmittance in the visible region. It is a measure of both volumetric and surface light scattering of the front and rear surfaces of a sample and can arise due to particles, roughness and index inhomogeneities in the material. Haze (%) was measured with a Haze-Gard Plus hazemeter in accordance with ASTM D1003 [44]. Change in haze (Δ*Haze*) was calculated via Eq 6,

$$\begin{array}{c} \begin{array}{c} \Delta Haze=Haze_{f}-Haze_{i}\;, \end{array} \end{array}$$ (6)

where *Haze*_*f*_ designates the value of haze (%) for a sample after a given exposure interval and *Haze*_*i*_ designates the value of haze (%) for the unexposed sample of the given grade of PET. Haze was measured for clear PET films, but not for white PET films because the TiO_2_-filled films are not optically transparent to visible light.

### Statistical modeling

MMR models were fit to the data following variable selection. Variable selection was conducted via a step-wise forward selection process using Akaike information criterion (AIC) [45]. AIC is a measure of relative quality between a group of models and accounts for the balance between complexity and goodness of fit of the models. Variable selection provides a rank-ordered set of predictors that are correlated to the response being modeled. The variable selection process for this study accounted for multiple responses, several predictors, and potential interactions between predictors. Material and exposure predictors were represented using indicator variables, which are terms that take value of 0 or 1 to indicate the absence or presence of a categorical value that is expected to affect the outcome of the model, respectively. Outliers resulting from measurement or experimental error were determined from exploratory data analysis and were removed before variable selection. Following the variable selection process, interaction terms based on weathering and materials science domain knowledge were added to the models (e.g. between photodose and moisture) to capture synergistic stressor effects and reveal differences in material performance. Interactions were limited to only two covariates, except for the case in which a strong rationale based on domain knowledge could be given for the addition of a three-covariate interaction term. The statistical significance of each predictor term was evaluated by its p-value with a 5% significance level [30]. Predictor terms with p-values larger than 0.05 were removed from the model in order of largest to smallest p-value.

In many cases, the relationships between predictors and responses is nonlinear. A natural spline was used for the photodose in this study to capture the nonlinear relationship between responses and the photodose predictor [46, 47]. The approach is borrowed from the generalized additive model (GAM), which provides a general framework for extending a standard linear model by allowing non-linear functions, commonly splines, of each predictor. This approach uses a linear combination of spline bases to estimate the relationship between predictors and responses [22]. A general basis model can be represented by Eq 7:

$$\begin{array}{c} f\left(X\right)=\sum_{q=0}^{Q}\beta_{q}\cdot b_{q}\left(X\right)=\beta_{0}+\beta_{1}b_{1}\left(X\right)+\beta_{2}b_{2}\left(X\right)+\ldots +\beta_{Q}b_{Q}\left(X\right), \end{array}$$ (7)

where *b*_1_(⋅), *b*_2_(⋅), …, *b*_*Q*_(⋅) are the basis functions, *Q* is the number of basis functions, and *β*_*q*_’s are coefficients. A cubic spline with *K* knots can be written in general form as shown in Eq 8:

$$\begin{array}{c} \begin{array}{cc} f(X) & =\beta_{0}+\beta_{1}X+\beta_{2}X^{2}+\beta_{3}X^{3}+\beta_{4}{\left(X-a_{1}\right)}_{+}^{3}\\ & +\beta_{5}{\left(X-a_{2}\right)}_{+}^{3}+\cdots +\beta_{K+3}{\left(X-a_{K}\right)}_{+}^{3}\;, \end{array} \end{array}$$ (8)

where ${\left(X-a\right)}_{+}^{3}={\left(X-a\right)}^{3}$ if *X* > *a* and 0 otherwise, and *a*_1_, …, *a*_*K*_ are knots. Knots define the break points or end points of each piecewise function of a spline. A natural cubic spline adds additional boundary constraints, namely that the function is linear beyond the boundary knots. This frees up four degrees of freedom (two constraints each in both boundary regions), which can be spent more profitably by placing more knots in the interior region. A natural cubic spline with *K* knots is represented by *K* basis functions as follows in Eq 9:

$$\begin{array}{c} \begin{array}{cc} & b_{1}\left(X\right)=1,\;b_{2}\left(X\right)=X,\\ & b_{k+2}\left(X\right)=d_{k}\left(X\right)-d_{K-1}\left(X\right),\;\;k=1,2,\ldots ,K, \end{array} \end{array}$$ (9)

where $d_{k}\left(X\right)=[{\left(X-a_{k}\right)}_{+}^{3}-{\left(X-a_{K}\right)}_{+}^{3}]/\left(a_{k}-a_{K}\right)$. In the context of MMR models, the natural spline terms become one column of the ***X*** matrix of predictors in Eq 3. The natural splines in this study included two internal knots. Knot positions were optimized to minimize the residual standard error of the models.

To validate the models, a training and testing framework was implemented using leave-one-out cross-validation (LOOCV) [30]. Cross validation is a model evaluation method that can be used to estimate the test error in terms of root mean square error (RMSE). The available set of observations is split into two parts, a training set and a validation set. The model is fit on the training set, and the fitted model is used to predict the response for the observations in the validation set. LOOCV involves using a single observation as the validation set and the remaining observations as the training set, and performing this process *n* times (for all data points). The model is fit on the first training set that contains all but one observation (*y*_1_) and a prediction (${\hat{y}}_{1}$) is made for this one observation. The mean squared error (MSE) is calculated by ${\text{MSE}}_{1}={\left(y_{1}-{\hat{y}}_{1}\right)}^{2}$. The process is repeated such that each observation is used as the validation set once and then *n* mean squared error (MSE_1_,…,MSE_*n*_) values are obtained. The root average of these *n* test errors is calculated by Eq 10:

$$\begin{array}{c} \text{RMSE}=\sqrt{\frac{1}{n}\sum_{i=1}^{n}{\left(y_{i}-{\hat{y}}_{i}\right)}^{2}}\;. \end{array}$$ (10)

In this study, the performance of each model was assessed through calculation of the RMSE for each of its responses across materials and exposures.

## Results

### Modeling of PET degradation under outdoor exposures

Clear and white grades of PET film were modeled and analyzed separately because they showed different degradation behavior, across all responses, under outdoor exposure. Periodic re-evaluation of unexposed samples was conducted and values were consistent across exposure intervals. No significant difference was observed between the fixed-mount and 2-axis tracking exposures during variable selection, so the exposure data for the two configurations was combined for each location. The difference between these locations, which is low moisture level in Arizona (Steppe) versus high moisture level in Florida (Monsoon), was accounted for with an indicator variable, denoted by *E*_1_. Natural splines were used to estimate the nonlinear relationship between each of the response variables and *UVA*_<360_ photodose. The grades of PET showed different degradation trends with increasing photodose and showed significant difference between one another during variable selection. Therefore, indicator variables were used to account for each grade of PET. Two indicator variables were used for the three clear grades of PET, denoted by *M*_1_ and *M*_2_. C1 was used as the reference material for clear PET, the one represented when all material grade indicator variables equal zero. Five indicator variables were used for six white grades of PET, denoted by *M*_3_ through *M*_7_. W5 was used as the reference material for white PET. The “Cover” exposure configuration variable was found to be significant during variable selection for clear PET films and one can observe that responses changed more slowly in this configuration. Therefore the remainder of the exposure configurations were grouped into “Open”, and an indicator variable, denoted by *E*_2_ was used to model the difference between “Open” and “Cover”. Interaction terms were allowed during variable selection as follows:

- Material grade indicator variables and *UVA*_<360_ photodose
- *E*_1_ and *UVA*_<360_ photodose
- *E*_2_ and *UVA*_<360_ photodose
- *E*_1_, *E*_2_, and *UVA*_<360_ photodose

After the stepwise variable selection process, the models were fit and coefficients were obtained. The final models are presented in subsequent subsections.

#### Model of clear PET degradation

Let Model C-Outdoor describe the outdoor degradation of clear PET films (Eq 11). The response variables were Δ*E*, Δ*Gloss*_60_, and Δ*Haze*. Internal knots were optimized at 66.2 *MJ*/*m*^2^ and 79.9 *MJ*/*m*^2^ to account for the non-linear relationship of the response variables and *UVA*_<360_.

$$\begin{array}{c} \begin{array}{cc} Y & =\beta_{00}+\beta_{01}M_{1}+\beta_{02}M_{2}+\beta_{03}E_{1}+\beta_{04}E_{2}\\ & +\sum_{q=1}^{Q}\beta_{q0}b_{q}\left(I\right)+\sum_{q=1}^{Q}\beta_{q1}b_{q}\left(I\right)M_{1}+\sum_{q=1}^{Q}\beta_{q2}b_{q}\left(I\right)M_{2}\\ & +\sum_{q=1}^{Q}\beta_{q3}b_{q}\left(I\right)E_{1}+\sum_{q=1}^{Q}\beta_{q4}b_{q}\left(I\right)E_{2}+\sum_{q=1}^{Q}\beta_{q5}b_{q}\left(I\right)E_{1}E_{2}. \end{array} \end{array}$$ (11)

Here, ***β***_***qi***_ terms are coefficients where *i* is an index, *I* represents *UVA*_<360_ photodose, *b*_*q*_(*I*), *q* = 1, 2, …, *Q*, are the basis functions of the natural spline for *UVA*_<360_ photodose with *q* = 0 representing the non-natural spline terms, *Q* is the number of spline bases, and ***Y***, *M*_1_, *M*_2_, *E*_1_, *E*_2_ are:

$$\begin{array}{c} Y=(\begin{array}{c} \Delta E,\Delta Gloss_{60},\Delta Haze \end{array}), \end{array}$$

$$\begin{array}{c} M_{1}=\{\begin{array}{cc} 1 & \text{if}\;\text{Material}\;=\;C2\\ 0 & \text{otherwise} \end{array}, \end{array}$$

$$\begin{array}{c} M_{2}=\{\begin{array}{cc} 1 & \text{if}\;\text{Material}\;=\;C3\\ 0 & \text{otherwise} \end{array}, \end{array}$$

$$\begin{array}{c} E_{1}=\{\begin{array}{cc} 1 & \text{if}\;\text{Location}\;=\;\text{Florida}\;\\ 0 & \text{if}\;\text{Location}\;=\;\text{Arizona}\; \end{array}, \end{array}$$

$$\begin{array}{c} E_{2}=\{\begin{array}{cc} 1 & \text{if}\;\text{Exposure}\;\text{Configuration}\;=\;\text{Cover}\\ 0 & \text{otherwise} \end{array}. \end{array}$$

**Fig 1:**
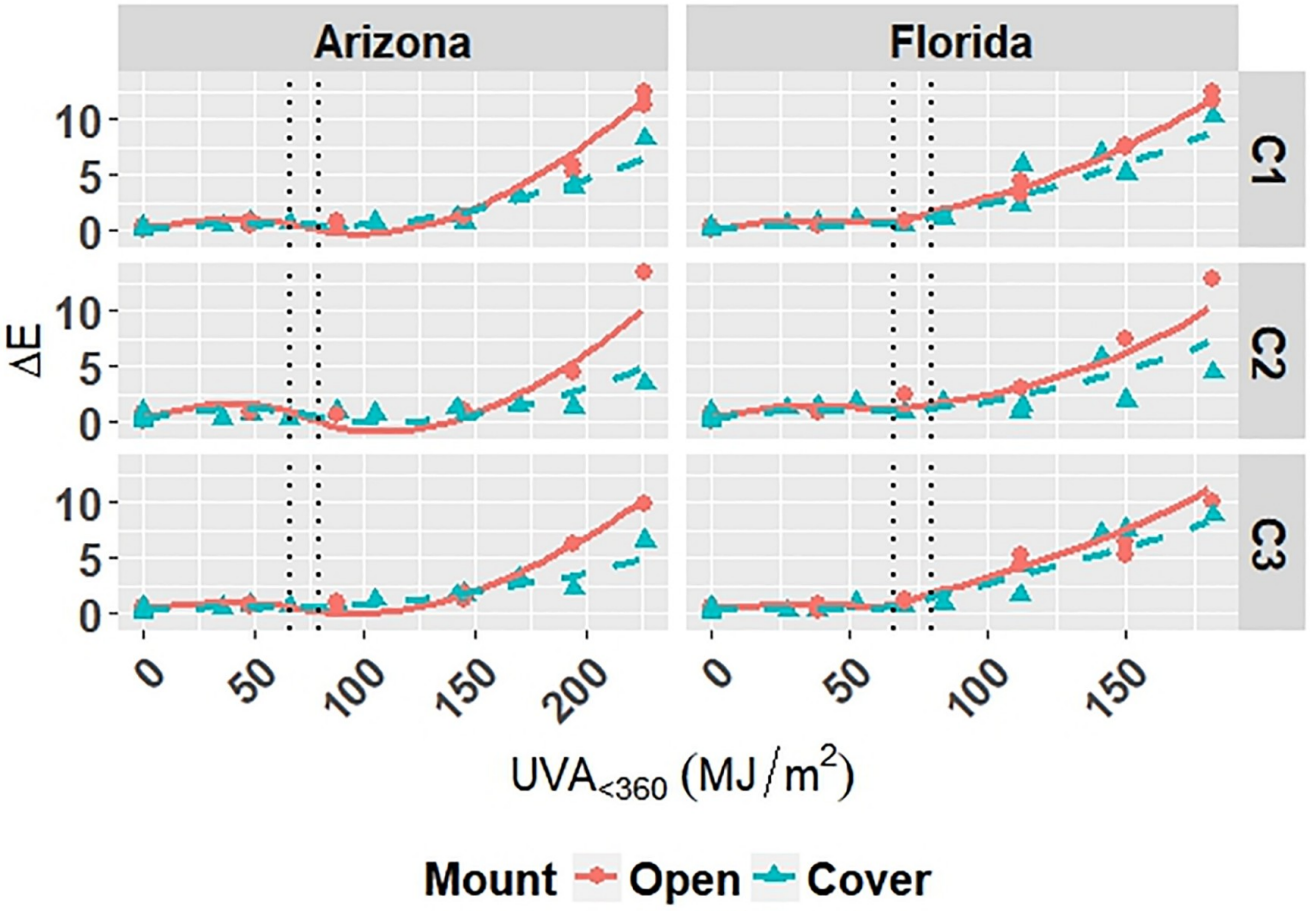
The change in Δ*E* clear grades of PET under outdoor exposures with the model curves superimposed on the data. Points represent the measured data and dashed lines represent the models. Vertical, dotted lines represent the location of natural spline knots.

**Fig 2:**
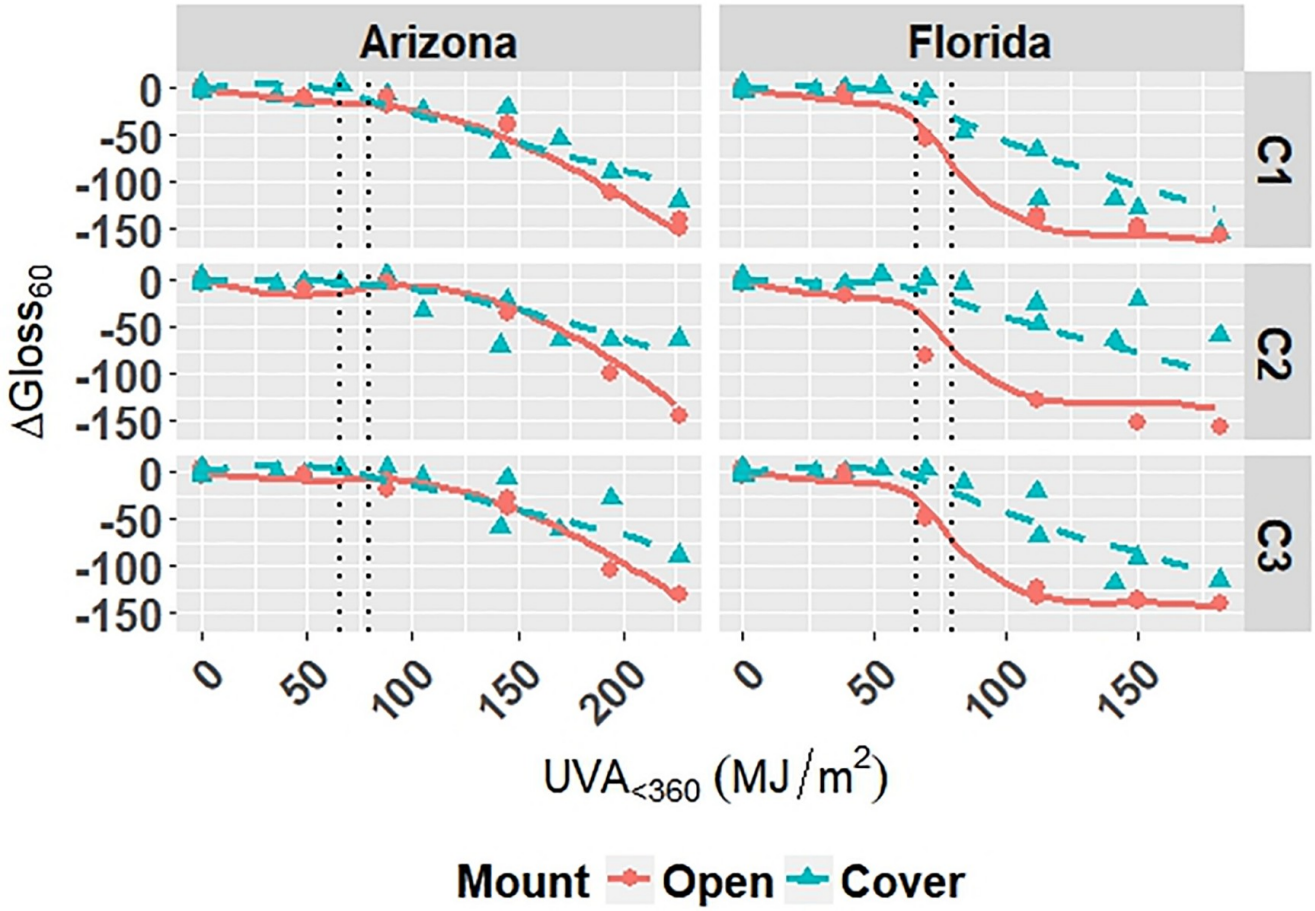
The change in Δ*Gloss*_60_ of clear grades of PET under outdoor exposures with the model curves superimposed on the data. Points represent the measured data and solid and dashed lines represent the models. Vertical, dotted lines represent the location of natural spline knots.

**Fig 3:**
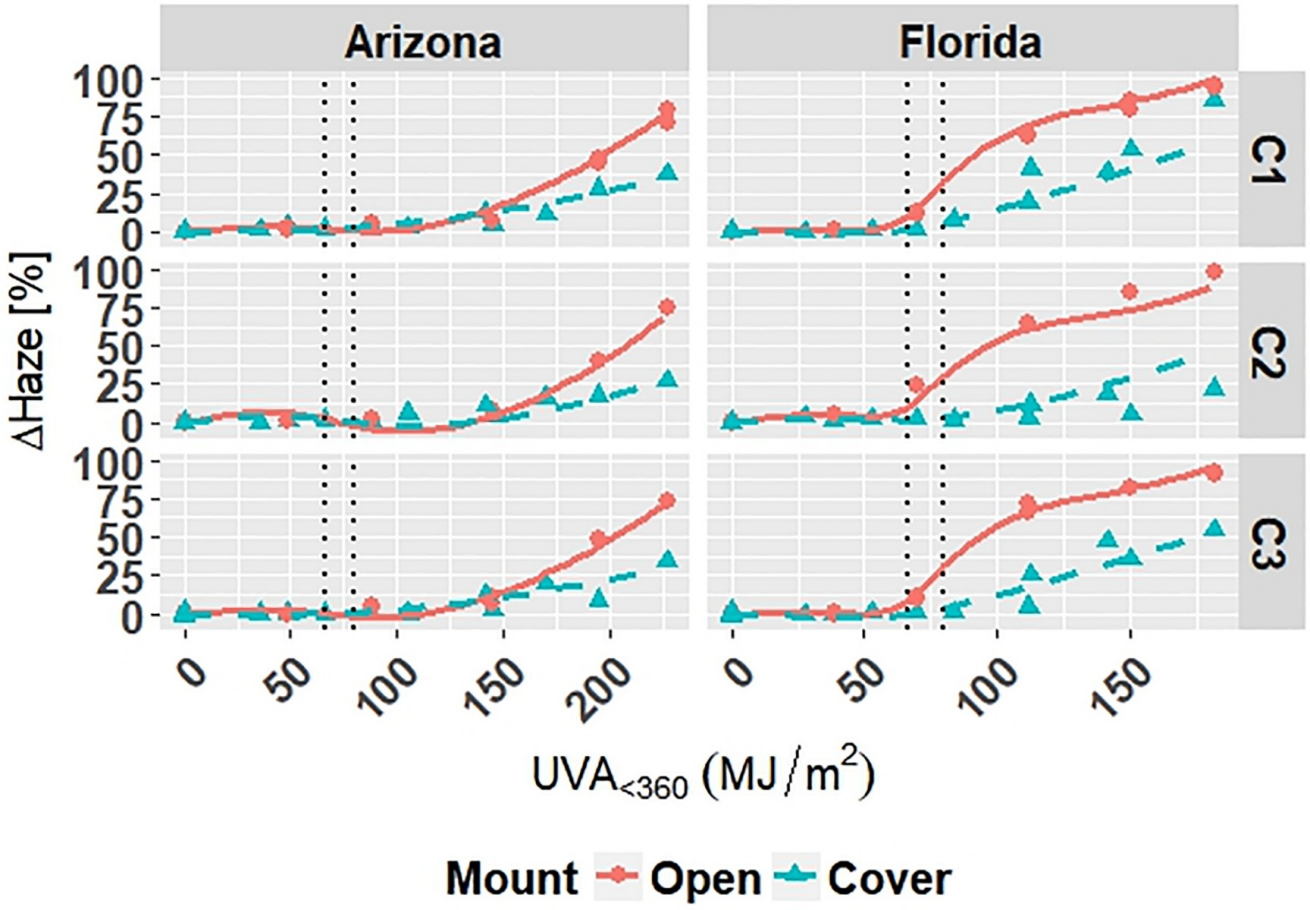
The change in Δ*Haze* of clear grades of PET under outdoor exposures with the model curves superimposed on the data. Points represent the measured data and solid and dashed lines represent the models. Vertical, dotted lines represent the location of natural spline knots.

Coefficients for Model C-Outdoor are given in Table 6. The model is superimposed on the observed values in Figs 1–3; outliers have been removed from the plots and subsequent analysis. The superimposed plots support the reliability of the models as predicted values trace the experimental data reasonably well. Diagnostic plots for the model are shown in Appendix B in S1 File. The residual errors for Model C-Outdoor are randomly distributed, which suggests that the model captures the relationship between predictors and responses well. The normal quantile-quantile plots for Model C-Outdoor show that the residuals have a heavy-tailed distribution meaning that there is more data located at the extremes and less data in the center of the distribution compared to a normal distribution, because the dataset contains numerous values at 0 *MJ*/*m*^2^ from repeated evaluation of unexposed films.

**Table 6:** Coefficients for Model C-Outdoor. *Y*_1_, *Y*_2_, and *Y*_3_ represent Δ*E*, Δ*Gloss*_60_, and Δ*Haze*, respectively. *q* is the natural spline basis function counter for *UVA*_<360_ photodose.

*q* = 0	***β*_00_**	***β*_01_**	***β*_02_**	***β*_03_**	***β*_04_**	***β*_05_**
*Y*_1_	-2.49	-1.32	1.27	-0.151	1.55	NA
*Y*_2_	18.4	16.7	-2.83	-2.70	-39.2	NA
*Y*_3_	-9.43	-11.5	1.21	-1.88	8.77	NA
*q* = 1	***β*_10_**	***β*_11_**	***β*_12_**	***β*_13_**	***β*_14_**	***β*_15_**
*Y*_1_	-3.04	-1.82	0.547	3.95	2.90	-1.87
*Y*_2_	-25.2	40.8	22.9	-142	-30.1	111
*Y*_3_	-8.38	-17.6	-3.24	78.1	15.9	-66.9
*q* = 2	***β*_20_**	***β*_21_**	***β*_22_**	***β*_23_**	***β*_24_**	***β*_25_**
*Y*_1_	18.2	1.95	-4.38	4.81	-9.21	-1.66
*Y*_2_	-198	-22.4	27.8	-118	152	79.3
*Y*_3_	98.2	22.7	-7.76	72.0	-62.1	-52.6
*q* = 3	***β*_30_**	***β*_31_**	***β*_32_**	***β*_33_**	***β*_34_**	***β*_35_**
*Y*_1_	25.5	-1.38	-4.43	5.68	-13.0	-1.18
*Y*_2_	-277	0.516	21.6	-95.3	136	52.7
*Y*_3_	154	4.59	-6.40	68.8	-92.7	-40.6

RMSE values obtained from LOOCV and adjusted *R*^2^ values for Model C-Outdoor are shown in Table 7. The adjusted *R*^2^ values show that the model explains 93.3%, 94.3%, and 96.4% of the variation in the data for Δ*E*, Δ*Gloss*_60_, and Δ*Haze*, respectively. The RMSE values show that the overall error of the model is small relative to the range of each response values; therefore, the model is a good overall fit to the data.

**Table 7:** Adjusted *R*^2^ and RMSE values for Model C-Outdoor. *Y*_1_, *Y*_2_, and *Y*_3_ are Δ*E*, Δ*Gloss*_60_, and Δ*Haze*, respectively.

	Adjusted *R*^2^	RMSE
*Y*_1_	0.933	0.220
*Y*_2_	0.943	3.45
*Y*_3_	0.964	1.01

#### Model of white PET degradation

Let Model W-Outdoor describe the outdoor degradation of white PET films (Eq 12) with coefficients given in Appendix C in S1 File. The response variables were Δ*E* and Δ*Gloss*_60_. Internal knots were optimized at 34.8 *MJ*/*m*^2^ and 69.8 *MJ*/*m*^2^ to account for the non-linear relationship of the response variables and *UVA*_<360_.

$$\begin{array}{c} \begin{array}{cc} Y & =\beta_{00}+\beta_{01}M_{3}+\beta_{02}M_{4}+\beta_{03}M_{5}+\beta_{04}M_{6}+\beta_{05}M_{7}+\beta_{06}E_{1}\\ & +\sum_{q=1}^{Q}\beta_{q0}b_{q}\left(I\right)+\sum_{q=1}^{Q}\beta_{q1}b_{q}\left(I\right)M_{3}+\sum_{q=1}^{Q}\beta_{q2}b_{q}\left(I\right)M_{4}\\ & +\sum_{q=1}^{Q}\beta_{q3}b_{q}\left(I\right)M_{5}+\sum_{q=1}^{Q}\beta_{q4}b_{q}\left(I\right)M_{6}+\sum_{q=1}^{Q}\beta_{q5}b_{q}\left(I\right)M_{7}\\ & +\sum_{q=1}^{Q}\beta_{q6}b_{q}\left(I\right)E_{1}. \end{array} \end{array}$$ (12)

Here, ***β***_***qi***_ terms are coefficients where *i* is an index, *I* represents *UVA*_<360_ photodose, *b*_*q*_(*I*), *q* = 1, 2, …, *Q*, are the basis functions of the natural spline for *UVA*_<360_ photodose with *q* = 0 representing the non-natural spline terms, *Q* is the number of spline bases, and ***Y***, *M*_3_, *M*_4_, *M*_5_, *M*_6_, *M*_7_, and *E*_1_ are:

$$Y=(\begin{array}{c} \Delta E,\Delta Gloss_{60} \end{array}),$$

$$\begin{array}{c} M_{3}=\{\begin{array}{cc} 1 & \text{if}\;\text{Material}\;=\;W1\\ 0 & \text{otherwise} \end{array}, \end{array}$$

$$\begin{array}{c} M_{4}=\{\begin{array}{cc} 1 & \text{if}\;\text{Material}\;=\;W2\\ 0 & \text{otherwise} \end{array}, \end{array}$$

$$\begin{array}{c} M_{5}=\{\begin{array}{cc} 1 & \text{if}\;\text{Material}\;=\;W3\\ 0 & \text{otherwise} \end{array}, \end{array}$$

$$\begin{array}{c} M_{6}=\{\begin{array}{cc} 1 & \text{if}\;\text{Material}\;=\;W4\\ 0 & \text{otherwise} \end{array}, \end{array}$$

$$\begin{array}{c} M_{7}=\{\begin{array}{cc} 1 & \text{if}\;\text{Material}\;=\;W6\\ 0 & \text{otherwise} \end{array}, \end{array}$$

$$\begin{array}{c} E_{1}=\{\begin{array}{cc} 1 & \text{if}\;\text{Location}\;=\;\text{Florida}\;\\ 0 & \text{if}\;\text{Location}\;=\;\text{Arizona}\; \end{array}. \end{array}$$

**Fig 4:**
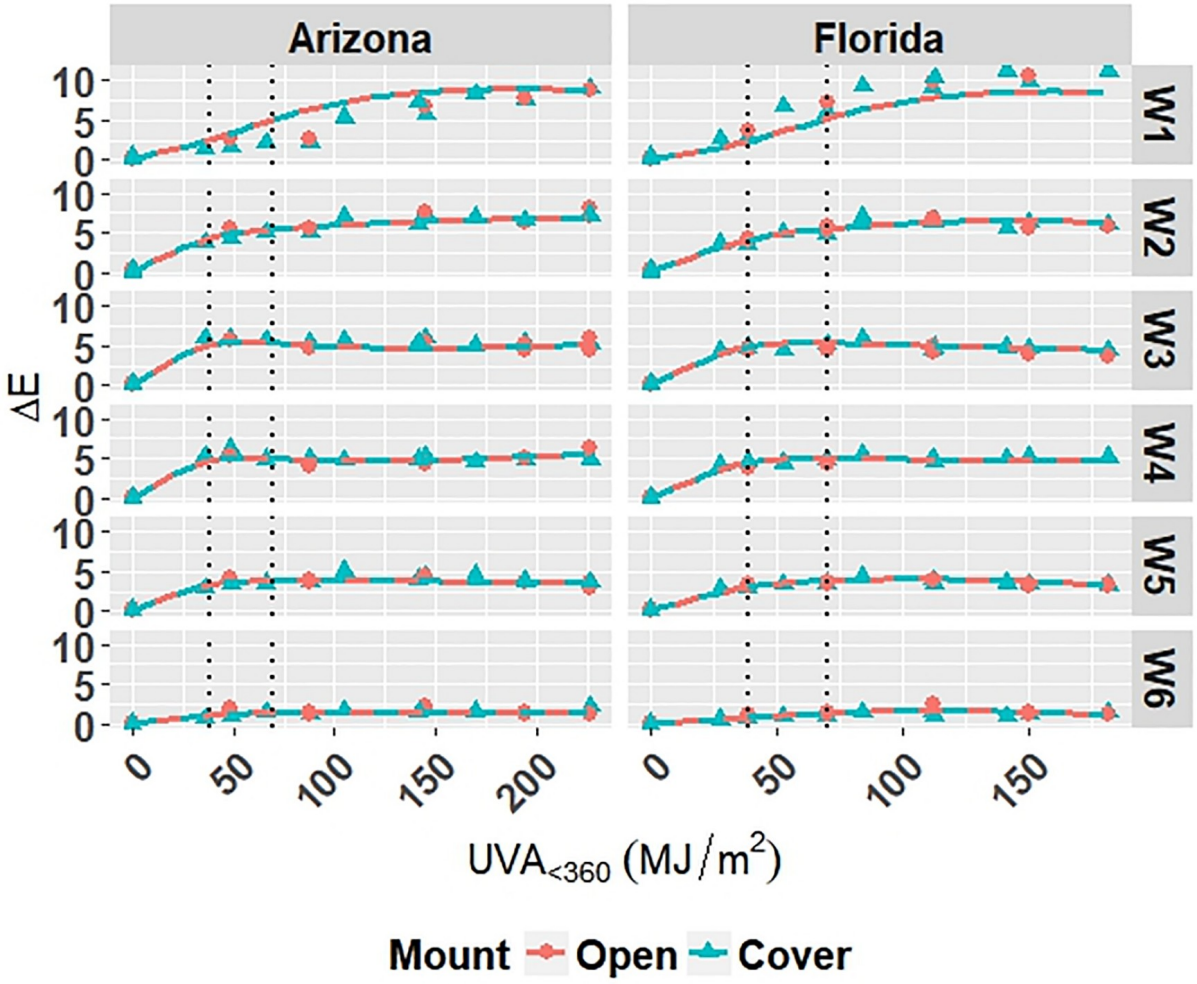
The change in Δ*E* for white grades of PET under outdoor exposures with the model curves superimposed on the data. Points represent the measured data and solid and dashed lines represent the models. Vertical, dotted lines represent the location of natural spline knots.

**Fig 5:**
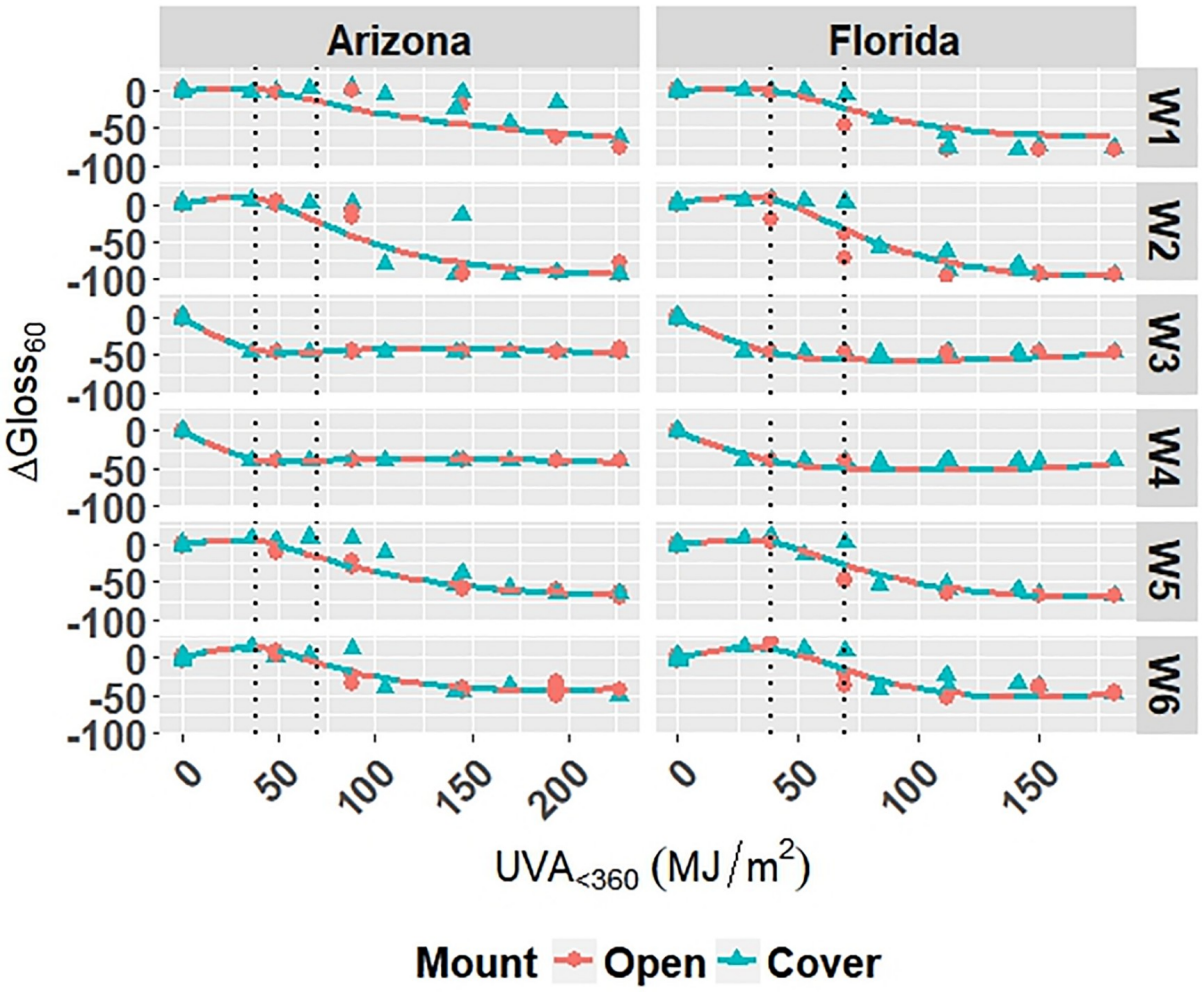
The change in Δ*Gloss*_60_ for white grades of PET under outdoor exposures with the model curves superimposed on the data. Points represent the measured data and solid and dashed lines represent the models. Vertical, dotted lines represent the location of natural spline knots.

The model is superimposed on the observed values in Figs 4 and 5. Diagnostic plots for the model are shown in Appendix C in S1 File. Model fit and diagnostic results for Model W-Outdoor were similar to those for Model C-Outdoor, which show that Model W-Outdoor is reliable.

RMSE values obtained from LOOCV and adjusted *R*^2^ values for Model W-Outdoor are shown in Table 8. The adjusted *R*^2^ values show that the model explains 96.1% and 90.7% of the variation in the data for Δ*E* and Δ*Gloss*_60_, respectively. The RMSE values show that the overall error of the model is small relative to the range of each response; therefore, the model is a good overall fit to the data.

**Table 8:** Adjusted *R*^2^ and RMSE values for Model W-Outdoor. *Y*_1_ and *Y*_2_ are Δ*E*, and Δ*Gloss*_60_, respectively.

	Adjusted *R*^2^	RMSE
*Y*_1_	0.961	0.139
*Y*_2_	0.907	2.22

### Modeling of PET degradation under accelerated exposures

Clear and white grades of PET film also showed different degradation response behavior under accelerated exposure, so they were modeled and analyzed separately. Periodic re-evaluation of unexposed samples was conducted and values were consistent across exposure intervals. The difference between wet and dry exposures was accounted for with an indicator variable, denoted as *E*_3_; wet exposures were defined as containing either condensing humidity or water spray such that the samples were in direct contact with water. Natural splines were used to estimate the nonlinear relationship between each of the response variables and *UVA*_<360_ photodose. The grades of PET showed different degradation trends with increasing photodose and significant difference between one another during variable selection. Therefore, indicator variables were used to account for each grade of PET. Chamber temperature was analyzed using its nominal values and was denoted in variable form as *E*_4_. Relative humidity was excluded from analysis because it was found insignificant during variable selection and the indicator variable representing direct moisture contact was found to more strongly account for the effect of hydrolysis. Interaction terms were allowed during variable selection as follows:

- Material grade indicator variables and *UVA*_<360_ photodose
- *E*_3_ and *UVA*_<360_ photodose
- *E*_4_ and *UVA*_<360_ photodose

After the stepwise variable selection process, the models were fit and coefficients were obtained. The final models are presented in subsequent sections.

#### Model of clear PET degradation

Let Model C-Accelerated describe the accelerated degradation of clear PET films (Eq 11) with coefficients given in Appendix D in S1 File. The response variables were Δ*E*, Δ*Gloss*_60_, and Δ*Haze*. Internal knots were optimized at 106.8 *MJ*/*m*^2^ and 108.0 *MJ*/*m*^2^ to account for the non-linear relationship of the response variables and *UVA*_<360_.

$$\begin{array}{c} \begin{array}{cc} Y & =\beta_{00}+\beta_{01}M_{1}+\beta_{02}M_{2}+\beta_{03}E_{3}+\beta_{04}E_{4}\\ & +\sum_{q=1}^{Q}\beta_{q0}b_{q}\left(I\right)+\sum_{q=1}^{Q}\beta_{q1}b_{q}\left(I\right)M_{1}+\sum_{q=1}^{Q}\beta_{q2}b_{q}\left(I\right)M_{2}\\ & +\sum_{q=1}^{Q}\beta_{q3}b_{q}\left(I\right)E_{3}+\sum_{q=1}^{Q}\beta_{q4}b_{q}\left(I\right)E_{4}. \end{array} \end{array}$$ (13)

Here, ***β***_***qi***_ terms are coefficients where *i* is an index, *I* represents *UVA*_<360_ photodose, *b*_*q*_(*I*), *q* = 1, 2, …, *Q*, are the basis functions of the natural spline for *UVA*_<360_ photodose with *q* = 0 representing the non-natural spline terms, *Q* is the number of spline bases, *E*_4_ is the chamber temperature (°C), and ***Y***, *M*_1_, *M*_2_, *E*_3_ are:

$$Y=(\begin{array}{c} \Delta E,\Delta Gloss_{60},\Delta Haze \end{array}),$$

$$\begin{array}{c} M_{1}=\{\begin{array}{cc} 1 & \text{if}\;\text{Material}\;=\;C2\\ 0 & \text{otherwise} \end{array}, \end{array}$$

$$\begin{array}{c} M_{2}=\{\begin{array}{cc} 1 & \text{if}\;\text{Material}\;=\;C3\\ 0 & \text{otherwise} \end{array}, \end{array}$$

$$\begin{array}{c} E_{3}=\{\begin{array}{cc} 1 & \text{if}\;\text{Wet}\;\text{Exposure}\;\text{Conditions}\\ 0 & \text{if}\;\text{Dry}\;\text{Exposure}\;\text{Conditions} \end{array}. \end{array}$$

**Fig 6:**
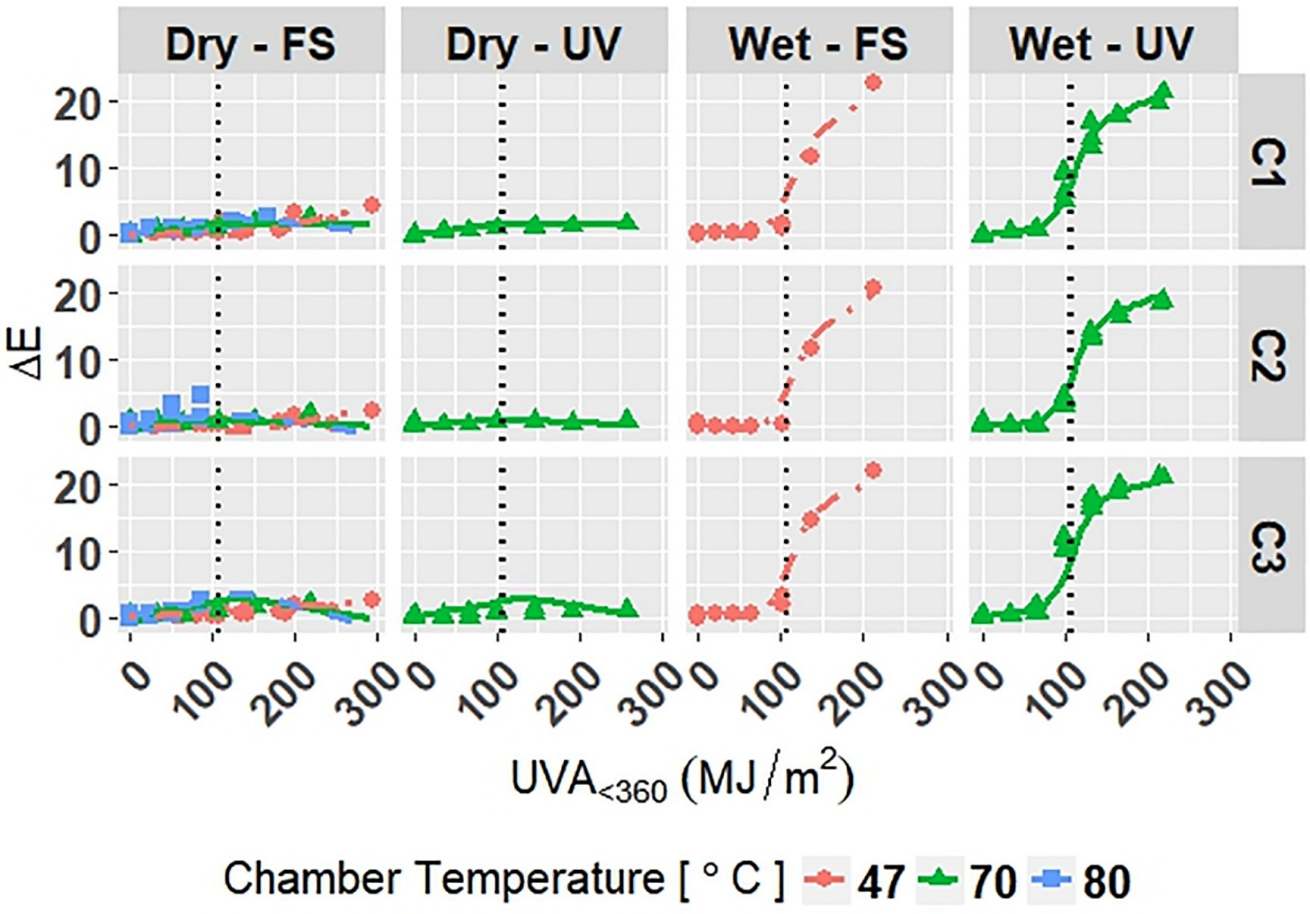
The change in Δ*E* of clear grades of PET under accelerated exposures with the model curves superimposed on the data. Points represent the measured data and solid and dashed lines represent the models. Vertical, dotted lines represent the location of natural spline knots.

**Fig 7:**
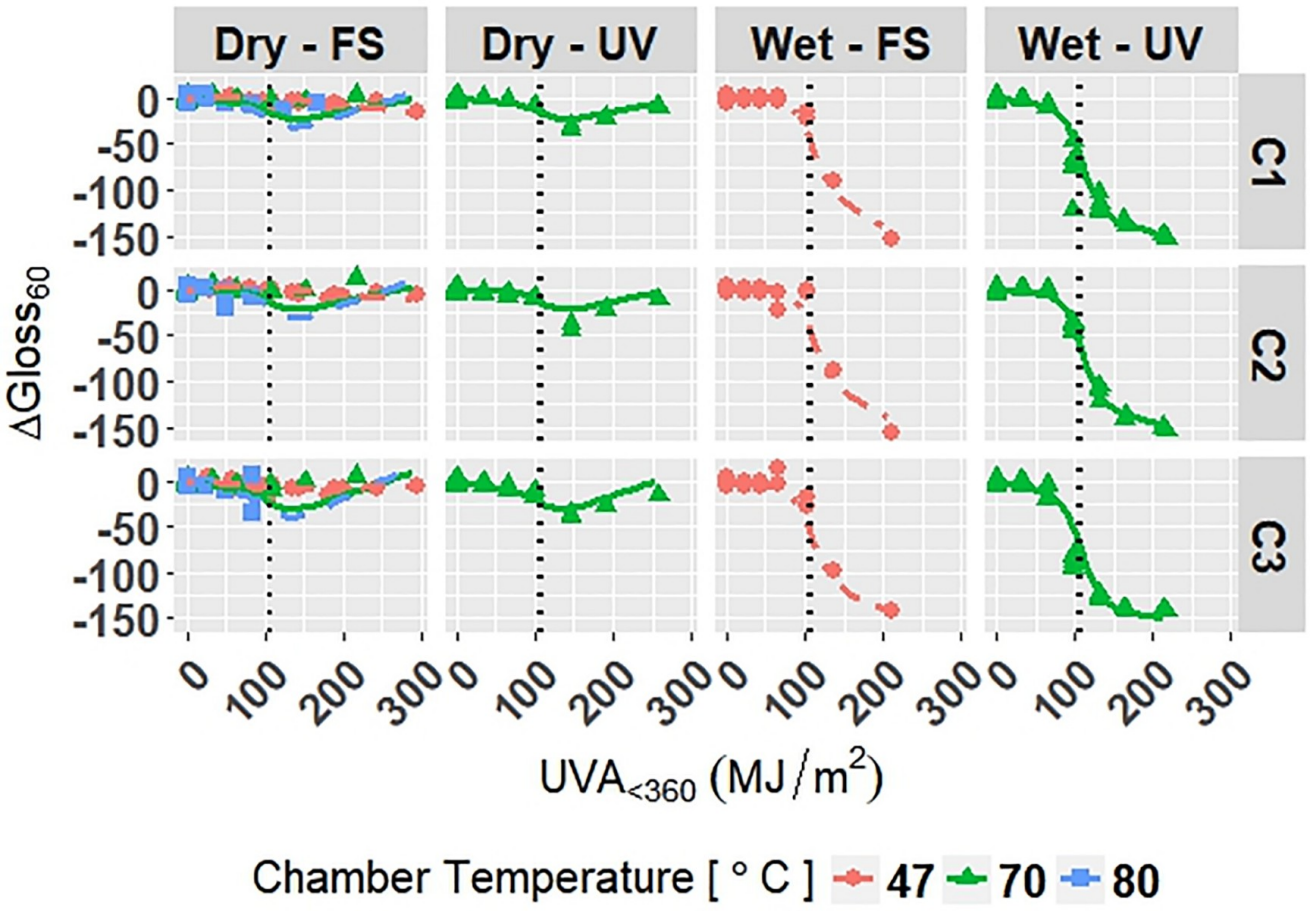
The change in Δ*Gloss*_60_ of clear grades of PET under accelerated exposures with the model curves superimposed on the data. Points represent the measured data and solid and dashed lines represent the models. Vertical, dotted lines represent the location of natural spline knots.

**Fig 8:**
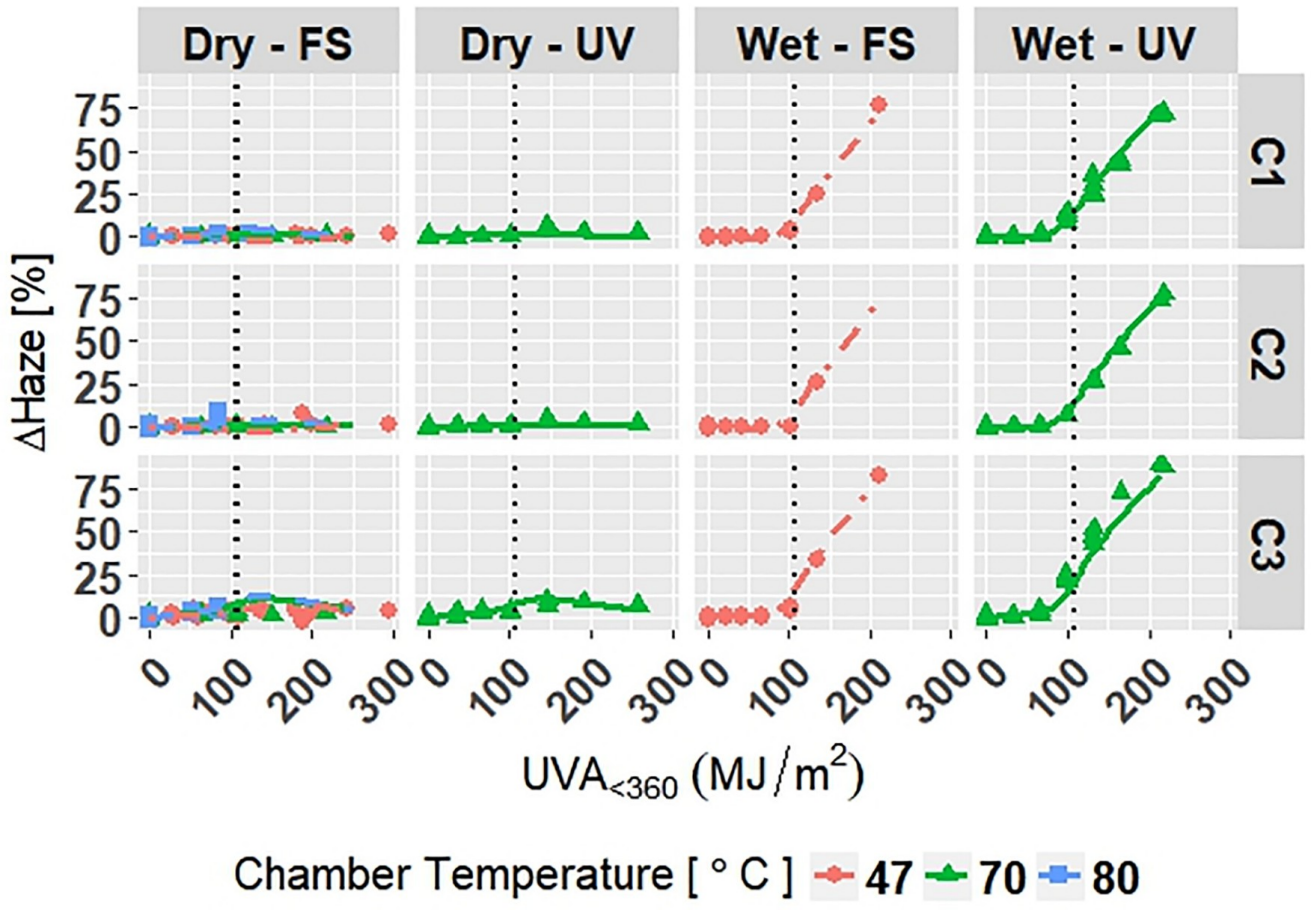
The change in Δ*Haze* of clear grades of PET under accelerated exposures with the model curves superimposed on the data. Points represent the measured data and solid and dashed lines represent the models. Vertical, dotted lines represent the location of natural spline knots.

The model is superimposed on the observed values in Figs 6–8. Diagnostic plots for the model are shown in Appendix D in S1 File. Model fit and diagnostic results for Model C-Accelerated were similar to those for the previous models, which show that Model C-Accelerated is reliable.

RMSE values obtained from LOOCV and adjusted *R*^2^ values for Model C-Accelerated are shown in Table 9. The adjusted *R*^2^ values show that the model explains 95.9%, 95.0%, and 96.7% of the variation in the data for Δ*E*, Δ*Gloss*_60_, and Δ*Haze*, respectively. The RMSE values show that the overall error of the model is small relative to the range of each response, therefore the model is a good overall fit to the data.

**Table 9:** Adjusted *R*^2^ and RMSE values for Model C-Accelerated. *Y*_1_, *Y*_2_, and *Y*_3_ are Δ*E*, Δ*Gloss*_60_, and Δ*Haze*, respectively.

	Adjusted *R*^2^	RMSE
*Y*_1_	0.959	0.368
*Y*_2_	0.950	3.91
*Y*_3_	0.967	1.11

#### Model of white PET degradation

Let Model W-Accelerated describe the accelerated degradation of white PET films (Eq 14) with coefficients given in Appendix E in S1 File. The response variables were Δ*E* and Δ*Gloss*_60_. Internal knots were optimized at 22.0 *MJ*/*m*^2^ and 65.8 *MJ*/*m*^2^ to account for the non-linear relationship of the response variables and *UVA*_<360_.

$$\begin{array}{c} \begin{array}{cc} Y & =\beta_{00}+\beta_{01}M_{3}+\beta_{02}M_{4}+\beta_{03}b_{q}\left(I\right)M_{5}+\beta_{04}M_{6}+\beta_{05}M_{7}\\ & +\sum_{q=1}^{Q}\beta_{q1}b_{q}\left(I\right)M_{3}+\sum_{q=1}^{Q}\beta_{q2}b_{q}\left(I\right)M_{4}+\sum_{q=1}^{Q}\beta_{q3}b_{q}\left(I\right)M_{5}\\ & +\sum_{q=1}^{Q}\beta_{q4}b_{q}\left(I\right)M_{6}+\sum_{q=1}^{Q}\beta_{q5}b_{q}\left(I\right)M_{7}+\sum_{q=1}^{Q}\beta_{q6}b_{q}\left(I\right)E_{3}\\ & +\sum_{q=1}^{Q}\beta_{q7}b_{q}\left(I\right)E_{4} \end{array} \end{array}$$ (14)

Here, ***β***_***qi***_ terms are coefficients where *i* is an index, *I* represents *UVA*_<360_ photodose, *b*_*q*_(*I*), *q* = 1, 2, …, *Q*, are the basis functions of the natural spline for *UVA*_<360_ photodose, *Q* is the number of spline bases with *q* = 0 representing the non-natural spline terms, *E*_4_ is the chamber temperature (°C), and ***Y***, *M*_3_, *M*_4_, *M*_5_, *M*_6_, *M*_7_, *E*_3_ are:

$$Y=(\begin{array}{c} \Delta E,\Delta Gloss_{60} \end{array}),$$

$$\begin{array}{c} M_{3}=\{\begin{array}{cc} 1 & \text{if}\;\text{Material}\;=\;W1\\ 0 & \text{otherwise} \end{array}, \end{array}$$

$$\begin{array}{c} M_{4}=\{\begin{array}{cc} 1 & \text{if}\;\text{Material}\;=\;W2\\ 0 & \text{otherwise} \end{array}, \end{array}$$

$$\begin{array}{c} M_{5}=\{\begin{array}{cc} 1 & \text{if}\;\text{Material}\;=\;W3\\ 0 & \text{otherwise} \end{array}, \end{array}$$

$$\begin{array}{c} M_{6}=\{\begin{array}{cc} 1 & \text{if}\;\text{Material}\;=\;W4\\ 0 & \text{otherwise} \end{array}, \end{array}$$

$$\begin{array}{c} M_{7}=\{\begin{array}{cc} 1 & \text{if}\;\text{Material}\;=\;W6\\ 0 & \text{otherwise} \end{array}, \end{array}$$

$$\begin{array}{c} E_{3}=\{\begin{array}{cc} 1 & \text{if}\;\text{Wet}\;\text{Exposure}\;\text{Conditions}\\ 0 & \text{if}\;\text{Dry}\;\text{Exposure}\;\text{Conditions} \end{array}. \end{array}$$

**Fig 9:**
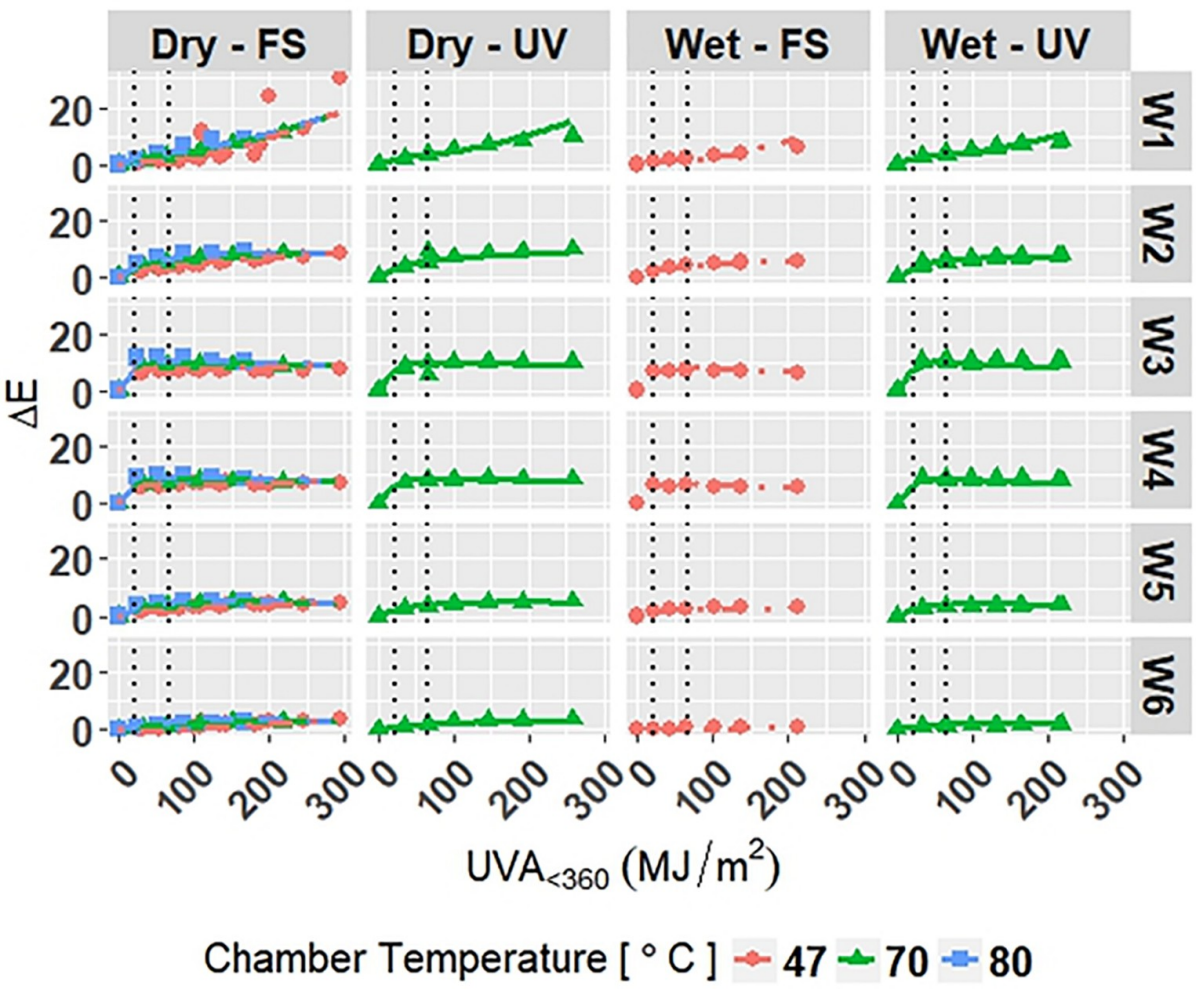
The change in Δ*E* for white grades of PET under accelerated exposures with the model curves superimposed on the data. Points represent the measured data and solid and dashed lines represent the models. Vertical, dotted lines represent the location of natural spline knots.

**Fig 10:**
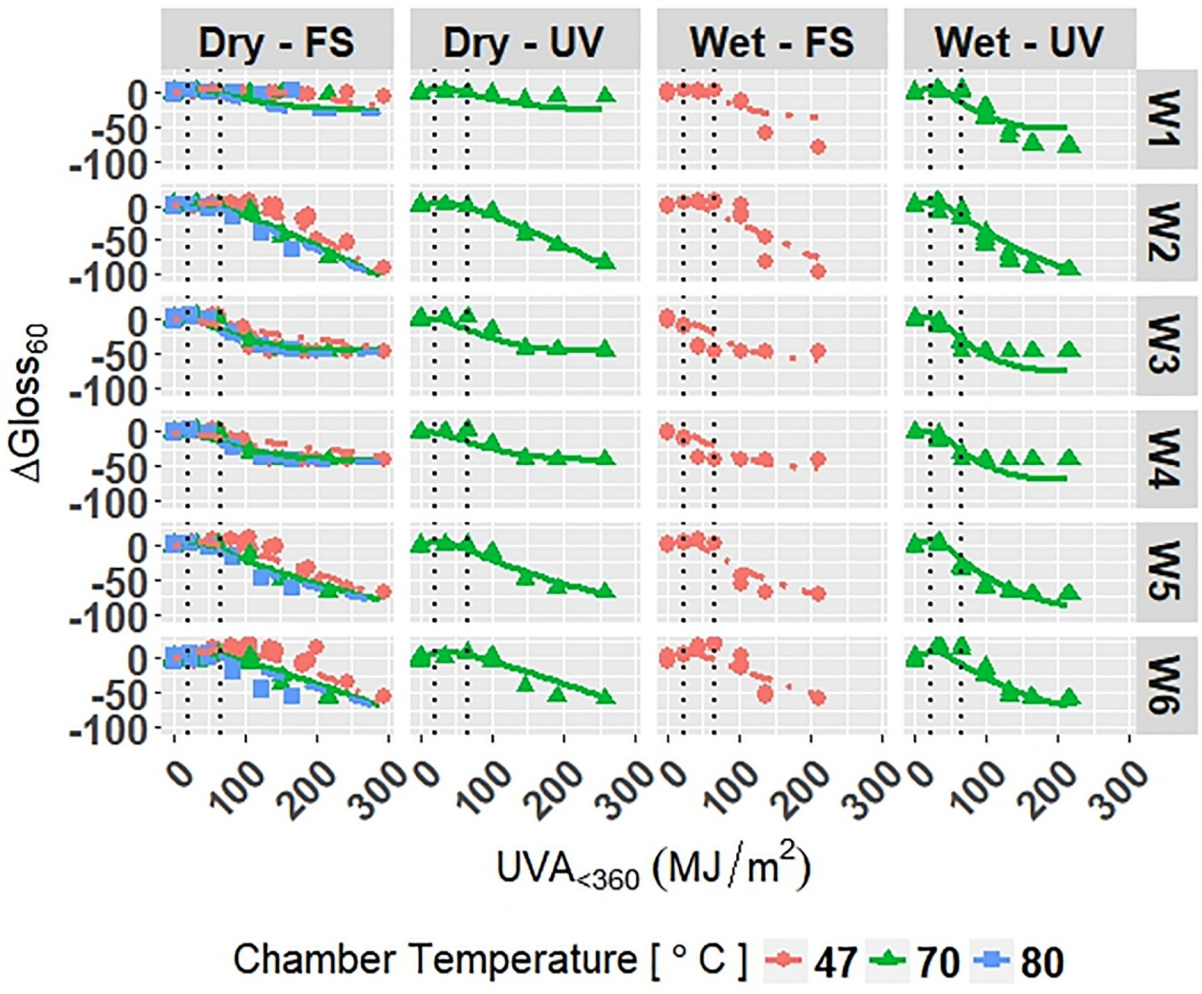
The change in Δ*Gloss*_60_ for white grades of PET under accelerated exposures with the model curves superimposed on the data. Points represent the measured data and solid and dashed lines represent the models. Vertical, dotted lines represent the location of natural spline knots.

The model is superimposed on the observed values in Figs 9 and 10. Diagnostic plots for the model are shown in Appendix E in S1 File. Model fit and diagnostic results for Model W-Accelerated were similar to those for the previous models, which show that Model W-Accelerated is reliable.

RMSE values obtained from LOOCV and adjusted *R*^2^ values are shown in Table 10 for Model W-Accelerated. The adjusted *R*^2^ values show that the model explains 95.1% and 88.2% of the variation in the data for Δ*E* and Δ*Gloss*_60_, respectively. The RMSE values show that the overall error of the model is small relative to the range of each response, therefore the model is a good overall fit to the data.

**Table 10:** Adjusted *R*^2^ and RMSE values for Model W-Accelerated. *Y*_1_ and *Y*_2_ are Δ*E* and Δ*Gloss*_60_, respectively.

	Adjusted *R*^2^	RMSE
*Y*_1_	0.951	0.339
*Y*_2_	0.882	4.45

## Discussion

### Role of stressors in PET degradation

All four models show that combinations of light, heat, and humidity caused change in the measured properties of PET that indicate photolysis and hydrolysis of the films. As degradation proceeded, Δ*E* (Figs 1, 4, 6 and 9) and Δ*Haze* (%) (Figs 3 and 8) generally increased. The stressor terms and exposure indicator terms that exacerbated degradation would generally carry positive coefficients and those that slowed degradation would carry negative coefficients (Table 6 and C.1-E.1). The opposite is true for Δ*Gloss*_60_ (Figs 2, 5, 7 and 10) because its value decreased as degradation proceeded. The magnitude of the coefficient is also useful in model interpretation; terms with coefficients that are larger magnitude show more impact on the degradation phenomena. The strong agreement between the model and the data suggests that photodose was most critical degradation stressor given that the models were fit considering photodose as the primary degradation stressor with the other stressors having synergistic interactions [7]. The strong fit of the photodose natural spline terms to the data suggests that PET follows a nonlinear degradation process.

Models C-Outdoor and C-Accelerated show that degradation of clear PET films was accelerated by moisture; the coefficients in Table 6 and D.1 for terms representing moisture stress (*E*_1_ and *E*_3_) are large in magnitude with the same sign as the response variable. The *E*_1_ coefficients from Model C-Outdoor (for example: 3.95 for Δ*E*, -142 for Δ*Gloss*_60_, and 78.1 for Δ*Haze* with *q* = 1, Table 6) show that degradation was strongly accelerated in Florida compared to Arizona. The Florida environment had higher relative humidity and higher precipitation, which increased moisture available to outdoor exposed films compared to Arizona. The *E*_2_ coefficients and those for the interaction of *E*_1_ and *E*_2_, in Table 6 showed that degradation was slower when the sample was under a borosilicate float glass cover-sheet. This suggests that direct water contact in the form of precipitation is primarily how moisture influences PET degradation, which is in agreement with Wypych [48]. This finding is supported by Model C-Accelerated, whose coefficients corresponding to *E*_3_ (wet exposures) showed that degradation proceeded more quickly under exposures that administered water spray or condensing humidity (FUV2, FUV3, Xe3). Direct water contact is more damaging because the water saturates the surface of the material and diffuses inward to drive hydrolytic reactions. Whereas airborne water in the form of humidity slowly arrives to the surface of the material. Model coefficients for Model W-Outdoor and W-Accelerated (Tables C.1 and E.1) show that this moisture driven effect was much less pronounced in white PET films.

The effect of temperature can be assessed through the Dry-FS exposures (Xe1, Xe2, Xe4, Xe5, and Xe6) in Models C-Accelerated and W-Accelerated. The coefficients in Tables D.1 and E.1 for temperature (*E*_4_) of both models show that increasing temperature increased the degradation rate of the materials. Increasing temperature accelerates the rates of the underlying chemical processes that drive photolytic and hydrolytic chemical processes [48].

Indicator variables accounting for the 2-axis solar tracking versus the fixed mount exposure were found to be insignificant during the variable selection process for the outdoor exposure models. Also, Figs 1–5 show no observable difference between the two types of exposure when the data from each is combined. This implies that the selection of 2-axis solar tracking or fixed mount for weathering testing does not change the degradation rate nor mechanism, but only the overall cumulative photodose.

### Effect of additives on PET degradation

Model coefficients (Table 6 and C.1-E.1) and plots of response variables versus photodose (Figs 1–10) can also be used to assess the impact of additives on PET degradation. The C3 grade of PET film contained Tinuvin 360 UV absorber and the W6 grade contained Tinuvin 1577, therefore it was expected that C3 and W6 would exhibit slower degradation rates in comparison to their counterparts due to protection against photolysis. Under outdoor exposure conditions without the protection of borofloat glass, C3 showed slower rate of color change and gloss loss, and similar rates of haze formation. Under accelerated exposure conditions, C3 exhibited a rate of color change between C1 and C2, and more rapid gloss loss and haze formation. These results suggest that the addition of Tinuvin 360 UV absorber to clear PET films only offers limited protection against the degradation of clear PET films for exposure doses relevant to solar applications, which are in agreement with Gok et al [7]. However, the slight benefit gained is most likely not worth the cost of the stabilizer. Tinuvin 360 is a less photostable UV absorber than Tinuvin 1577, and the use of less stable UV absorbers offers reduced photolytic protection [49].

The W6 grade of PET exhibited the overall slowest rates of color change and gloss loss under outdoor exposure conditions of the white PET films. W6 also showed the slowest rate of color change under accelerated exposure conditions. This result suggests that the addition of Tinuvin 1577 UV absorber to W6 was highly functional across many exposure conditions; however, the strong performance of W6 is convoluted in that it also contains the highest PVC of TiO_2_. TiO_2_ is known to mitigate the weathering induced degradation of polymeric materials [48]. TiO_2_ acts as a UV stabilizer by scattering damaging radiation away from the surface of the film. The TiO_2_ scatters light due to the contrast in index of refraction of PET (1.57) and TiO_2_ (2.6). Light scattering increases the optical path length in the material and decreases the optical penetration depth of the light into the material, keeping it to the near-surface layer [50–52]. The W3 and W4 grades of PET film undergo relatively large change in color and gloss after the first exposure step and then remain fairly constant across all exposures. This could indicate that the films reach their failure points rather abruptly in comparison to the others. The W3 and W4 grades have the lowest initial values of *Gloss*_60_, therefore relatively less gloss loss is necessary to reach minimum Δ*Gloss*_60_. Ignoring the unique behavior of the W3 and W4 films, increasing PVC of TiO_2_ of the films yielded overall increased protection from color change and gloss loss. The degradation of TiO_2_-containing films was observed to be relatively unaffected by direct moisture contact, unlike that of the clear PET films. This result suggests that TiO_2_ aids in preventing moisture from initiating hydrolysis reactions.

Hydrolytic stabilizers were added to W2 and W5 in the form of cyclohexanedimethanol. Cyclohexanedimethanol is a precursor comonomer to PET and is known to improve the hydrolysis resistance of PET under processing conditions [53]. The effect of these stabilizers cannot be assessed, because all of the white PET films with PVC values greater than 1% showed resistance to the effects of moisture across all exposures.

### Comparison of MMR models to physics-based models

The equation for Arrhenius-type modeling of photolytic degradation is shown in Eq 15:

$$\begin{array}{c} \begin{array}{c} ln\left(k\right)=\frac{-E_{a}}{R}(\frac{1}{T})+ln\left(A\right) \end{array} \end{array}$$ (15)

where *s* is the rate constant, *E*_*a*_ is activation energy, *R* is the gas constant, *T* is temperature, *A* is the pre-exponential factor (a constant).

**Fig 11:**
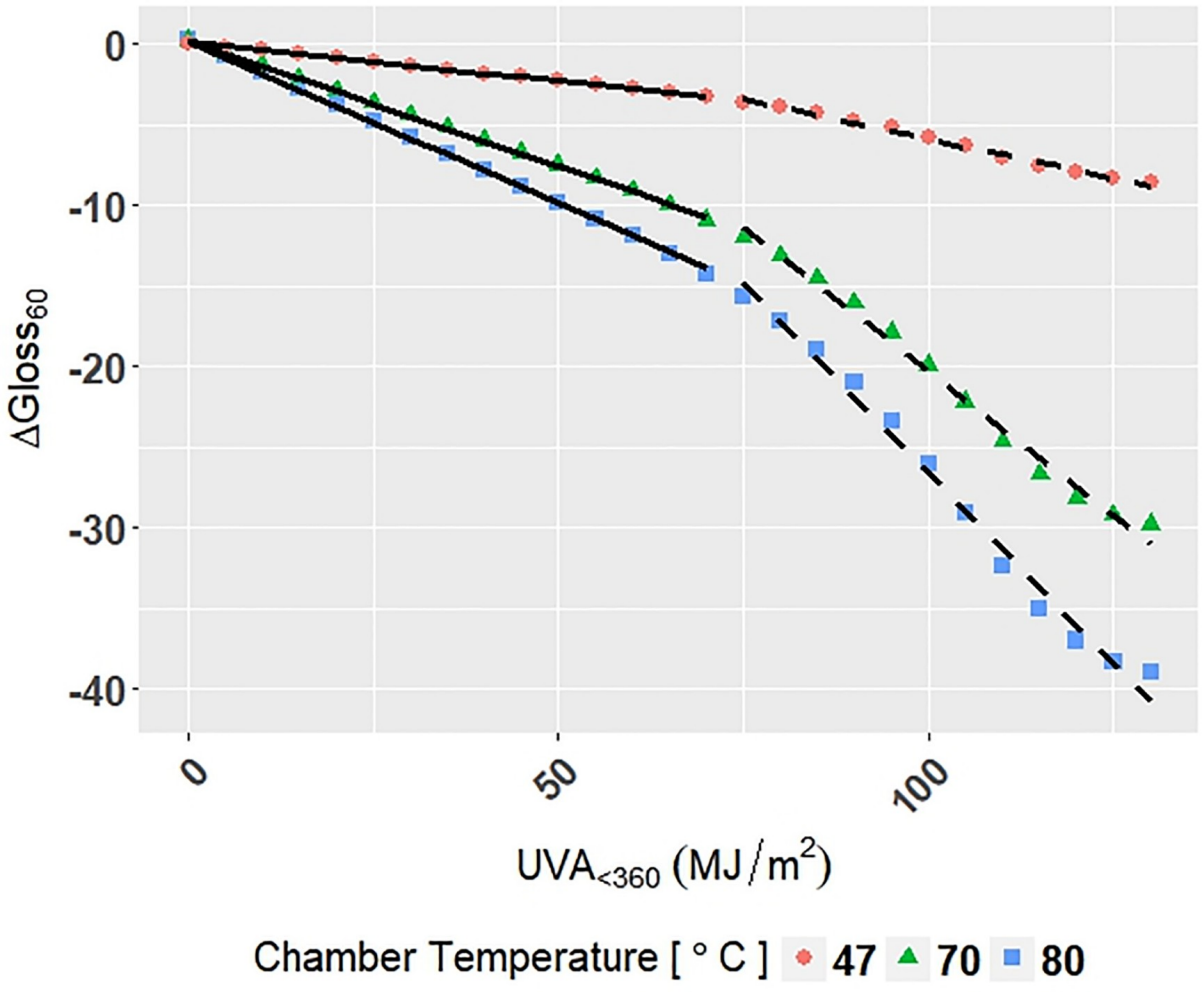
The change in Δ*Gloss*_60_ for the C3 grade of PET under Dry-FS accelerated exposure conditions with the Arrhenius models superimposed on the data. Points represent the measured data, the solid line represents the Arrhenius model for Section 1, and the solid and dashed line represents the Arrhenius model for Section 2.

The curves from Model C-Accelerated for Δ*Gloss*_60_ of the C3 grade of PET under Dry-FS accelerated exposure conditions at different temperatures (47°, 70°, and 80°) were fit with Eq 15 to compare the MMR models to the photolytic Arrhenius-type model. The curves of Δ*Gloss*_60_ were split into two sections to highlight change in the rate of gloss loss with increasing time under stress. Section 1 was defined from 0 to 70 *MJ*/*m*^2^ of *UVA*_<360_ photodose, and Section 2 was defined from 75 to 130 *MJ*/*m*^2^ (Fig 11).

Values of the Arrhenius rate constant *k* were obtained for both sections from a simple linear fit between Δ*Gloss*_60_ and *UVA*_<360_ photodose that accounted for each temperature. Activation energies were calculated by fitting Eq 15 to the rate constant (*k*) values for both Section 1 and Section 2. The adjusted-*R*^2^ of the fits were 0.999 for Section 1 and 0.998 for Section 2. Activation energies of 0.319 kcal/mol and 0.331 kcal/mol were obtained for Section 1 and Section 2, respectively. These values are within the range of activation energies of various engineering thermoplastics, 0-5 kcal/mol, presented by Pickett [26]. The agreement shows compatibility between the data-driven MMR model and the physics-based model approaches. The activation energies show that there was a 12% increase in activation energy from Section 1 to Section 2 of the *UVA*_<360_ photodose axis. A single Arrhenius model alone would not have captured this change-point phenomenon, because deviations from the Arrhenius law are observed with non-linear temperature dependence. Examples of deviation from expected Arrhenius behavior include glass transition in polymers and other glass-forming materials, and enzyme reactions [54, 55]. In contrast, natural splines accurately describe the non-linear behavior and can be used to perform Arrhenius-type analysis.

### Cross-correlation of accelerated exposure to outdoor exposure

**Fig 12:**
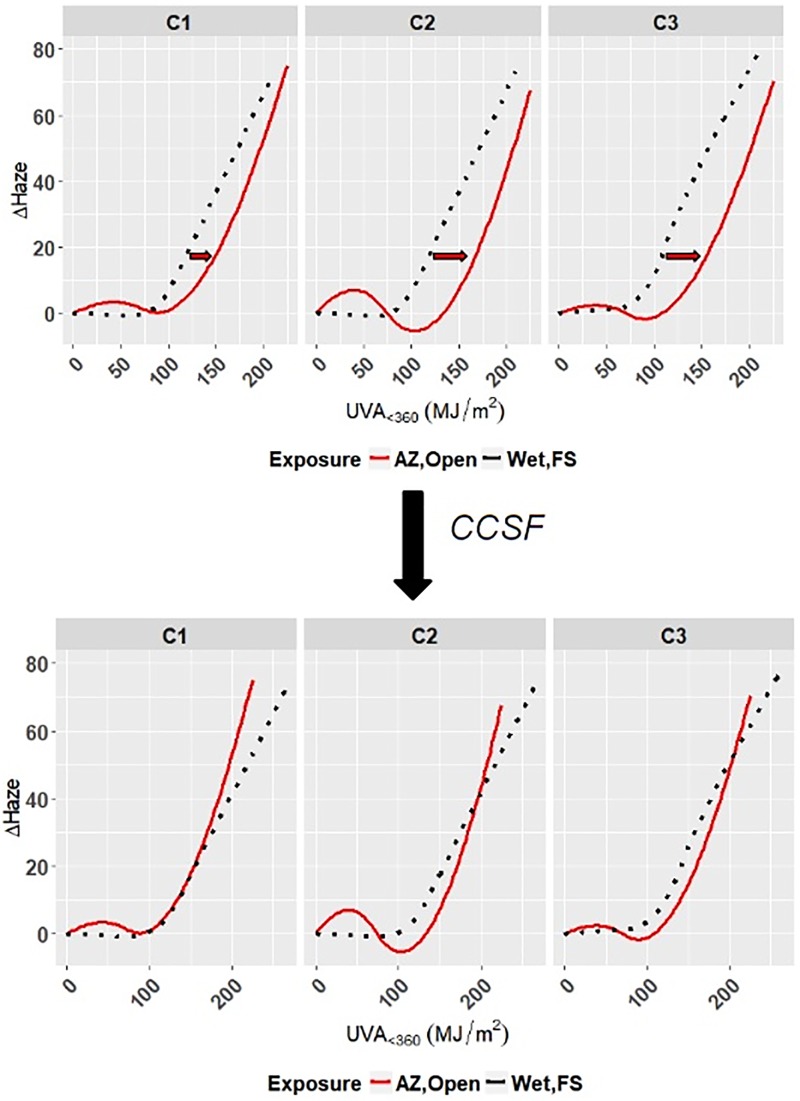
Example of scaling the model curve for an accelerated exposure (Wet-FS) by cross-correlation scale factor to minimize the RMSE with a given outdoor exposure model curve (Arizona-Open).

Cross-correlation of outdoor and accelerated exposures involves finding the accelerated exposure(s) that best represent the changes in properties exhibited under the various outdoor exposures. The task of finding the best match between accelerated and outdoor exposures was approached by determining the cross-correlation scale factor (CCSF) of the model curves of accelerated exposures (Wet-UV, Wet-FS, Dry-UV, Dry-FS) relative to the model curves of outdoor exposures (Arizona-Open, Arizona-Cover, Florida-Open, Florida-Cover) for clear and white PET separately. The CCSF between accelerated and outdoor model curves is the value that can be used to scale the photodose or time axis of the accelerated model curve such that it best approximates the outdoor model curve. Fig 12 provides a visual example of the result of cross-correlation in which the RMSE between the accelerated and outdoor model curves were minimized and CCSF was applied.

*I*_*in*_ and *I*_*out*_ are denoted to be the photodose for the accelerated and outdoor exposures, respectively. Let *Y*_*in*_ = *f*_1_(*I*_*in*_) and *Y*_*out*_ = *f*_2_(*I*_*out*_) be the accelerated and outdoor model functions, respectively. The photodose *I*_*in*_ was rescaled to $I_{in}^{⋆}=I_{in}/c$, where *c* is the value of CCSF. Based on the ordinary least square method, the sum of square errors were minimized, $L\left(c\right)=\sum {\left(Y_{in_{i}}^{⋆}-Y_{out_{i}}\right)}^{2}$, where $Y_{in_{i}}^{⋆}=f_{1}\left(I_{in}^{⋆}\right)$, and then the least square estimate of *c* was obtained, denoted as *c*^⋆^ (Eq 16).

$$\begin{array}{c} c^{⋆}=\text{arg}\;{\text{min}}_{c}\;L\left(c\right)=\text{arg}\;{\text{min}}_{c}\sum {\left(Y_{in_{i}}^{⋆}-Y_{out_{i}}\right)}^{2} \end{array}$$ (16)

A CCSF value less than one indicates that the films degraded more quickly under accelerated exposure conditions, whereas a CCSF value greater than one indicates that degradation proceeded more slowly under accelerated exposure conditions. The Dry-FS model curves included Dry-FS47, Dry-FS70, and Dry-FS80 that account for the curves for different chamber temperatures.

Cross-correlation scale factors and associated RMSE values are shown in Tables 11 and 12. Note that RMSE is calculated by $\sqrt{\sum {\left(Y_{in_{i}}-Y_{out_{i}}\right)}^{2}/n}$. Table 12 shows that the same best match exposure, CCSF, and RMSE values were obtained for the “Open” and “Cover” variants of Arizona and Florida outdoor exposure of white PET films. This is expected because there is little difference between the model curves for “Open” and “Cover” exposure variants for white PET films (Figs 4 and 5). Tables 11 and 12 show that model curves for accelerated exposure that induce direct moisture contact (Wet-UV and Wet-FS) were found to best correspond to the majority of model curves for outdoor exposure. This indicates that direct moisture contact is a key factor of accelerated weathering testing to obtain results that are comparable to outdoor exposure results. The CCSF values obtained for Δ*Gloss*_60_ and Δ*Haze* for the Florida-Open model curves for clear PET (1.38 and 1.71) are greater than one, which represents that in these cases the films experienced change more quickly under outdoor exposure than under accelerated exposure. This relationship was also observed across all responses and exposures for white PET. This indicates that accelerated exposure conditions could be made more aggressive to obtain reliable results more quickly for materials, responses, and exposures in which CCSF values are greater than one.

**Table 11:** Cross-correlation scale factors and RMSE values for the best match indoor sub-model for each outdoor sub-model for clear PET.

Best Match (CCSF, RMSE)	Δ*E*	Δ*Gloss*_60_	Δ*Haze*
Arizona, Open:	Wet-FS (0.55, 0.704)	Wet-UV (0.64, 10.7)	Wet-FS (0.79, 6.42)
Arizona, Cover:	Wet-UV (0.45, 0.513)	Wet-UV (0.57, 14.8)	Wet-UV (0.60, 4.10)
Florida, Open:	Wet-UV (0.69, 1.12)	Wet-UV (1.38, 8.36)	Wet-FS (1.71, 5.08)
Florida, Cover:	Wet-UV (0.64, 1.002)	Wet-FS (0.94, 15.1)	Wet-UV (0.93, 4.16)

**Table 12:** Cross-correlation scale factors and RMSE values for the best match indoor sub-model for each outdoor sub-model for white PET.

Best Match (CCSF, RMSE)	Δ*E*	Δ*Gloss*_60_
Arizona-Open:	Wet-FS (1.69, 1.55)	Dry-UV/Dry-FS70 (1.70, 10.8)
Arizona-Cover:	Wet-FS (1.69, 1.55)	Dry-UV/Dry-FS70 (1.70, 10.8)
Florida-Open:	Wet-FS (1.66, 1.60)	Wet-FS (1.69, 10.6)
Florida-Cover:	Wet-FS (1.66, 1.60)	Wet-FS (1.69, 10.6)

## Conclusions

Development and interpretation of data-driven MMR models have proven useful for analysis of the change in response variables (color, gloss, and haze) for clear and white PET films and the underlying roles of exposure stressors and material additives. Clear and white PET films must be considered separately when modeling response behavior, due to the difference in the evolution of their degradation responses. The use of natural splines to account for the nonlinear degradation phenomena was proven useful, which was reflected by high adjusted *R*^2^ values for the models, low model RMSE, and agreement with physics-based modeling.

Results of model interpretation has provided insights that are informative for future weathering experiments and materials developers. For outdoor exposures, solar tracking was found to have no effect on the degradation rate nor on the degradation mechanism for PET. Photodose was found to be the primary degradation stressor for PET. Direct moisture contact (rain, water spray, or condensation) was found to increase the degradation rate of PET and was found to be the primary source of hydrolysis stress instead of airborne moisture (humidity). Increasing temperatures were found to increase the degradation rate of PET, but not change the degradation mechanism. Increasing the concentration of TiO_2_ was found to generally decrease the degradation rate of PET and prevent hydrolytic degradation. The Tinuvin 360 UV absorber was found to be ineffective in clear PET films for the given solar application.

Cross-correlation of outdoor and accelerated exposures was achieved via optimization of cross-correlation scale factors, which allow one to select a best match accelerated exposure for a given outdoor exposure and compare between the two exposures. Interpretation of cross-correlation results revealed that direct moisture contact is a key factor for reliable accelerated weathering testing and has provided a quantitative method to determine when accelerated exposure results can be made more aggressive.

## Supporting information

S1 File Supporting information Appendices. Appendices include figures of data, model fits, summary statistics of the models that were omitted from the main text.
(PDF)
