# Supplementary material for: Multivariate multiple regression models of poly(ethylene-terephthalate) film degradation under outdoor and multi-stressor accelerated weathering exposures
Source: PLoS One. 2018 Dec 20;13(12):e0209016. doi: 10.1371/journal.pone.0209016 (PMC6301690; doi:10.1371/journal.pone.0209016)
Supplement: S1 File — Appendices include figures of data, model fits, summary statistics of the models that were omitted from the main text. (PDF) [file pone.0209016.s001.pdf]

Supporting Information: Multivariate multiple regression models of poly(ethylene-terephthalate) film degradation under outdoor and multi-stressor accelerated weathering exposures

Devin A. Gordon[1,2], Wei-Heng Huang[2], David M. Burns[3], Roger H. French[2,1], Laura S. Bruckman[*,2,¤],

1 Department of Macromolecular Science and Engineering, Case Western Reserve University, Cleveland, Ohio, United States
2 SDLE Research Center, Department of Materials Science and Engineering, Case Western Reserve University, Cleveland, Ohio, United States
3 3M Company, Maplewood, Minnesota, United States

¤SDLE Research Center, Department of Materials Science and Engineering, Case Western Reserve University, Cleveland, Ohio, United States
* lsh41@case.edu

Supporting Information

Figures of data, model fits, summary statistics of the models omitted from the main text are given.

Statistical computation and modeling was performed using R [1] and R Studio [2]. R packages used for analysis and plotting include: *ggplot2* [3], *qtlmt* [4], *splines* [5], *car* [6], *MASS* [7], *gam* [8–12], *caret* [13], and *kgc* [14].

Appendix A

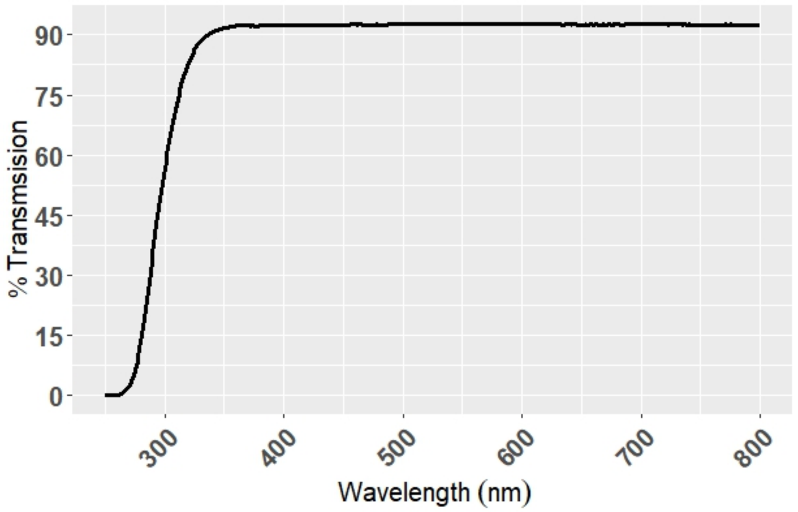

Fig A.1. Transmission spectrum of the borosilicate glass used in this study.

Appendix B

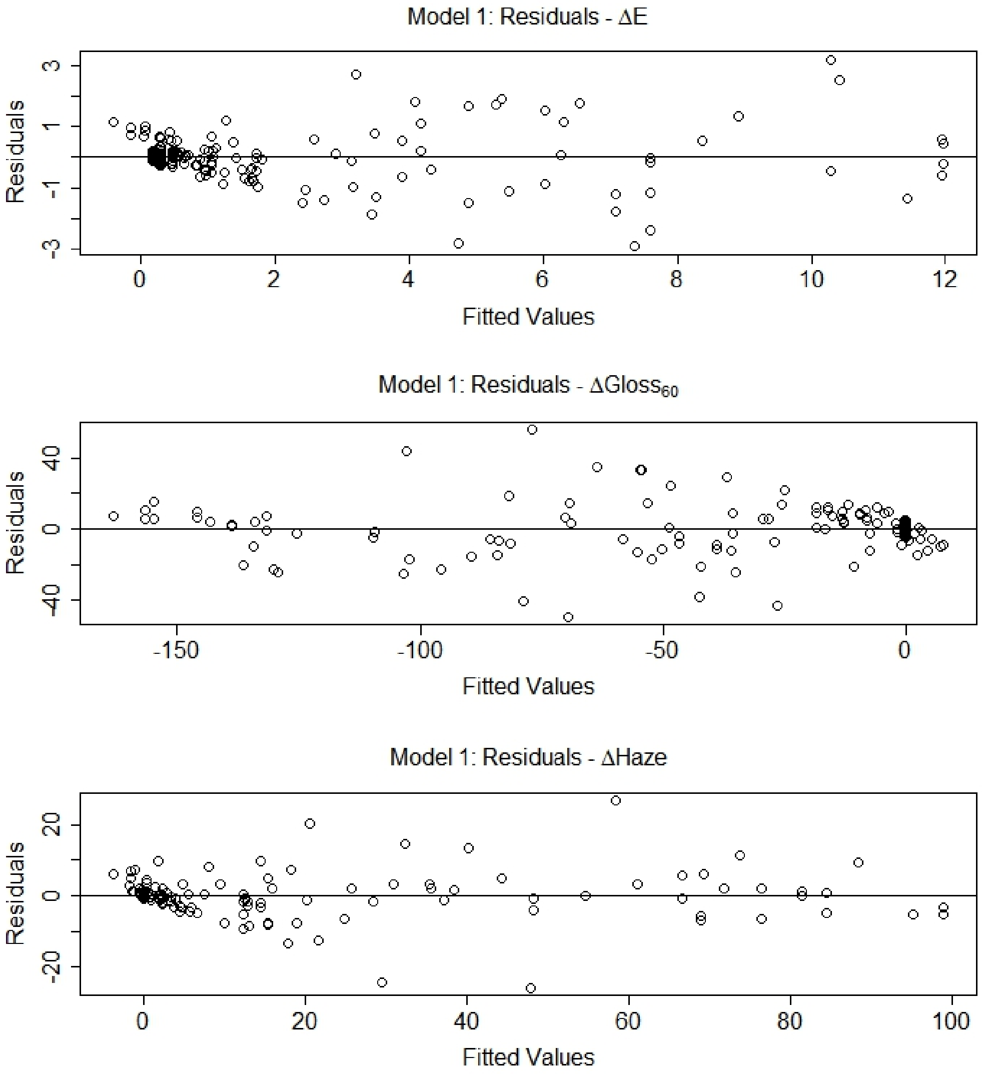

Fig B.1. Residuals versus fitted values diagnostic plots for ΔE, $\Delta Gloss_{60}$, and $\Delta Haze$ for Model 1.

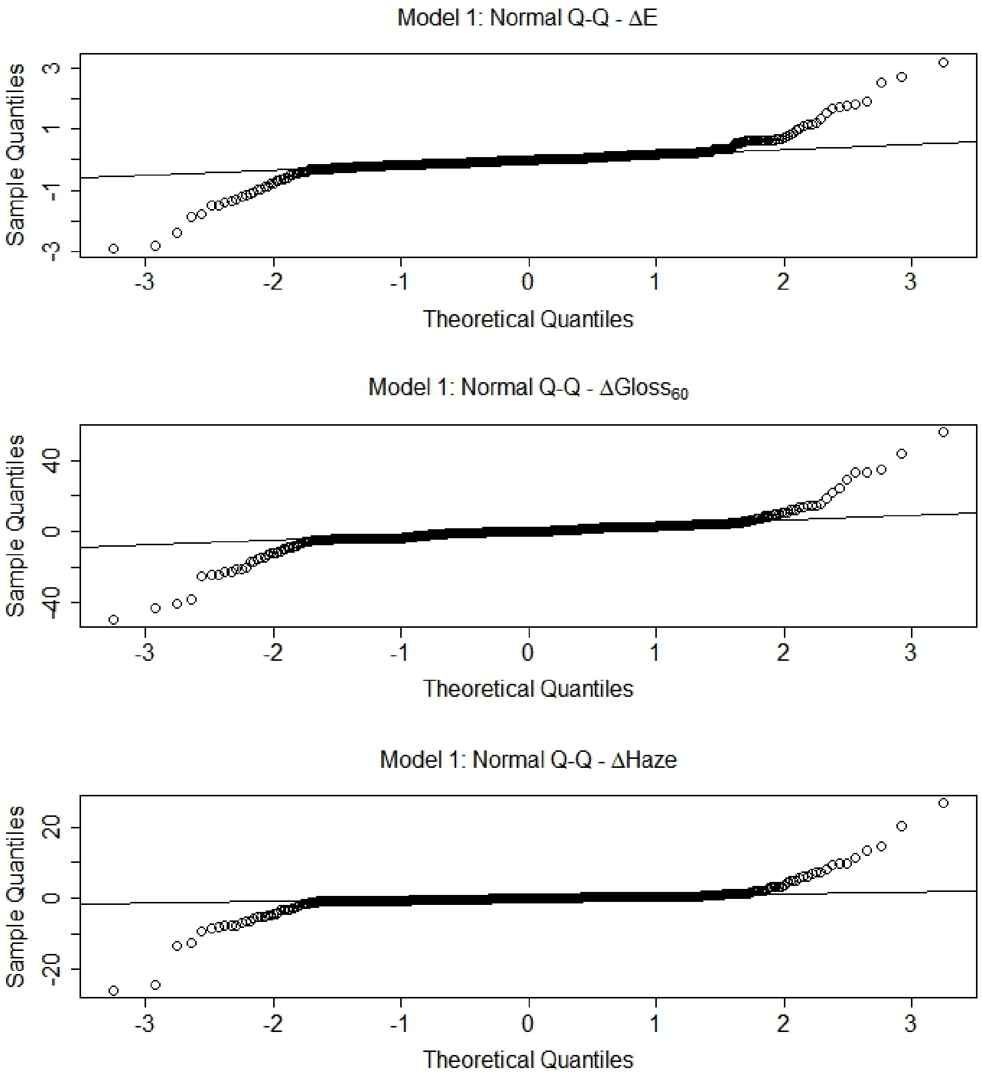

Fig B.2. Quantile-Quantile values diagnostic plots for ΔE, $\Delta Gloss_{60}$, and $\Delta Haze$ for Model 1.

Appendix C

Table C.1. Coefficients for Model W-Outdoor. Y_1 and Y_2 represent ΔE and $\Delta Gloss_{60}$, respectively. q is the natural spline basis function counter for $UVA_{<360}$ photodose.

$q=0$	β_{00}	β_{01}	β_{02}	β_{03}	β_{04}	β_{05}	β_{06}
Y_1	0.07	0.15	0.13	0.04	0.03	-0.01	-0.0002
Y_2	0.004	-0.01	-0.04	-0.02	-0.02	0.0003	0.008
$q=1$	β_{10}	β_{11}	β_{12}	β_{13}	β_{14}	β_{15}	β_{16}
Y_1	2.47	5.83	2.01	-0.57	-0.54	-1.42	0.90
Y_2	-59.5	15.2	-31.3	45.8	45.7	4.00	-27.7
$q=2$	β_{20}	β_{21}	β_{22}	β_{23}	β_{24}	β_{25}	β_{26}
Y_1	6.85	3.66	3.68	-0.57	2.80	-4.26	-0.63
Y_2	-50.7	4.95	-19.5	-36.0	-24.81	28.10	-7.18
$q=3$	β_{30}	β_{31}	β_{32}	β_{33}	β_{34}	β_{35}	β_{36}
Y_1	1.72	5.96	2.88	0.53	1.24	-0.75	-0.83
Y_2	-73.1	7.29	-28.8	50.7	52.7	21.4	6.33

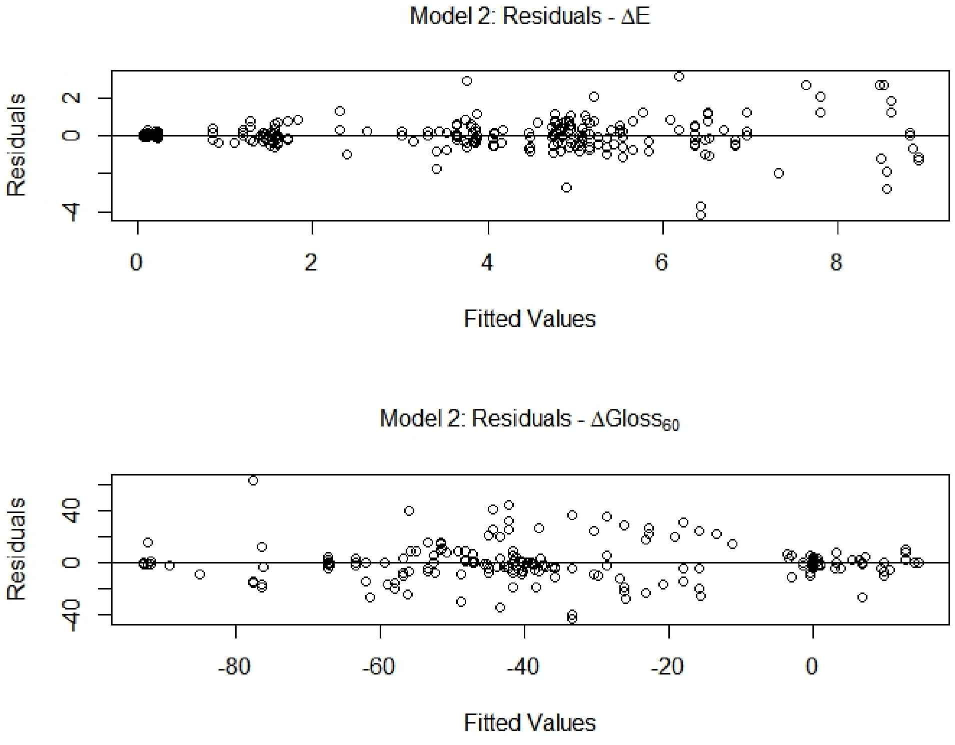

Fig C.1. Residuals versus fitted values diagnostic plots for ΔE and $\Delta Gloss_{60}$ for Model W-Outdoor.

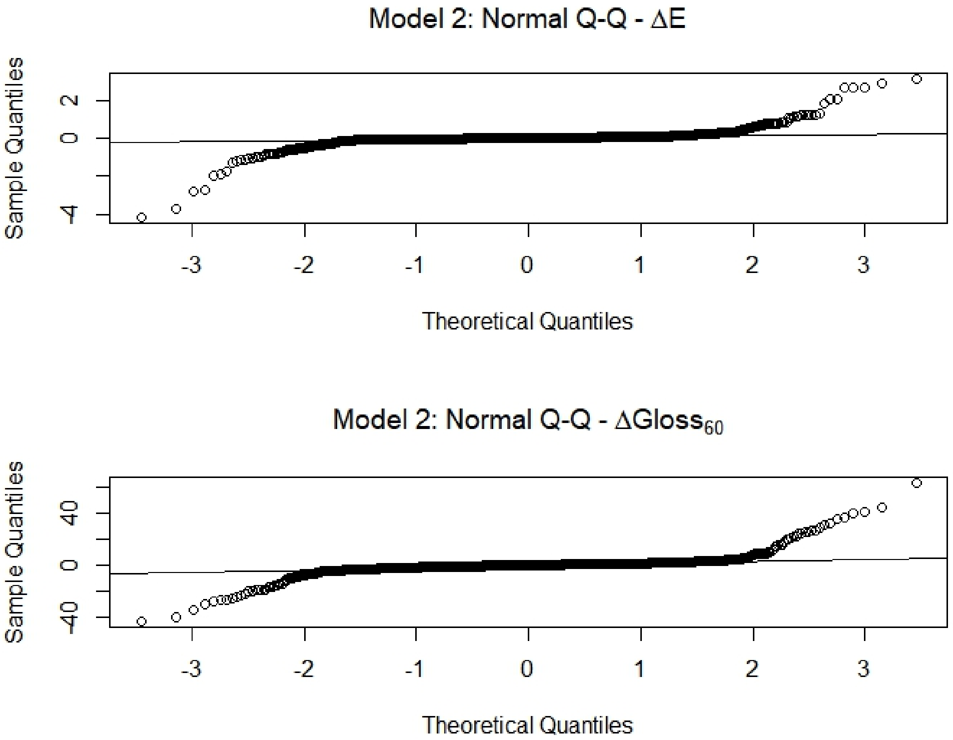

Fig C.2. Quantile-Quantile values diagnostic plots for ΔE and $\Delta Gloss_{60}$ for Model W-Outdoor.

Appendix D

Table D.1. Coefficients for Model C-Accelerated. Y_1, Y_2, and Y_3 represent ΔE, $\Delta Gloss_{60}$,and $\Delta Haze$, respectively. q is the natural spline basis function counter for $UVA_{<360}$ photodose.

$q = 0$	$\boldsymbol{\beta_{00}}$	$\boldsymbol{\beta_{01}}$	$\boldsymbol{\beta_{02}}$	$\boldsymbol{\beta_{03}}$	$\boldsymbol{\beta_{04}}$
Y_1	-1.11	-0.27	0.34	-0.12	0.03
Y_2	12.0	2.59	-3.62	-2.36	-0.23
Y_3	-2.45	-0.16	1.68	-0.41	0.05
$q = 1$	$\boldsymbol{\beta_{10}}$	$\boldsymbol{\beta_{11}}$	$\boldsymbol{\beta_{12}}$	$\boldsymbol{\beta_{13}}$	$\boldsymbol{\beta_{14}}$
Y_1	-2.77	-0.39	1.07	14.6	0.05
Y_2	35.6	-3.42	-4.71	-100	-0.80
Y_3	-11.7	0.40	8.46	28.7	0.18
$q = 2$	$\boldsymbol{\beta_{20}}$	$\boldsymbol{\beta_{21}}$	$\boldsymbol{\beta_{22}}$	$\boldsymbol{\beta_{23}}$	$\boldsymbol{\beta_{24}}$
Y_1	-2.26	-0.89	0.71	15.7	0.05
Y_2	34.6	0.08	-5.76	-111.88	-0.79
Y_3	-11.3	0.39	9.22	41.2	0.18
$q = 3$	$\boldsymbol{\beta_{30}}$	$\boldsymbol{\beta_{31}}$	$\boldsymbol{\beta_{32}}$	$\boldsymbol{\beta_{33}}$	$\boldsymbol{\beta_{34}}$
Y_1	1.19	-0.59	0.19	17.0	-0.009
Y_2	8.01	-2.40	0.89	-118	-0.30
Y_3	-5.58	0.88	6.42	54.2	0.08

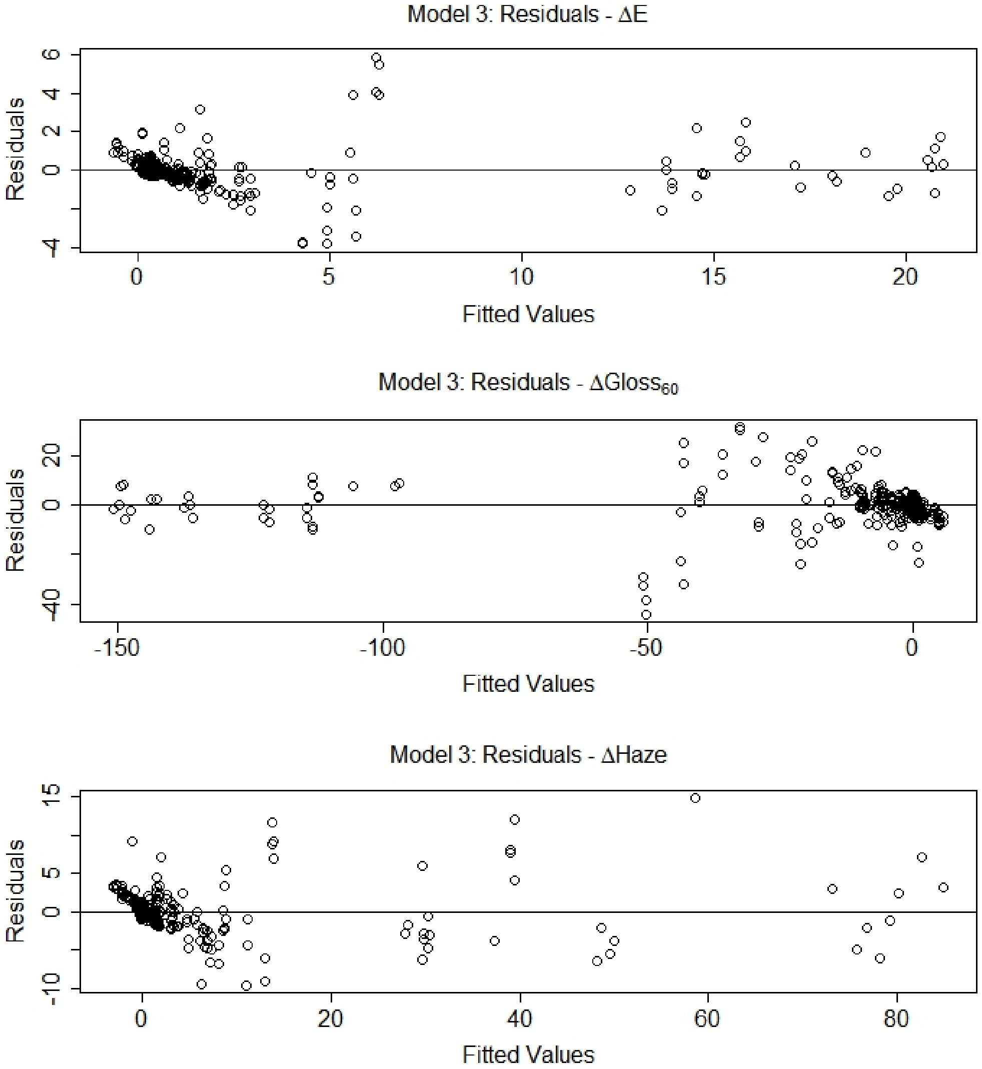

Fig D.1. Residuals versus fitted values diagnostic plots for ΔE, $\Delta Gloss_{60}$, and $\Delta Haze$ for Model C-Accelerated.

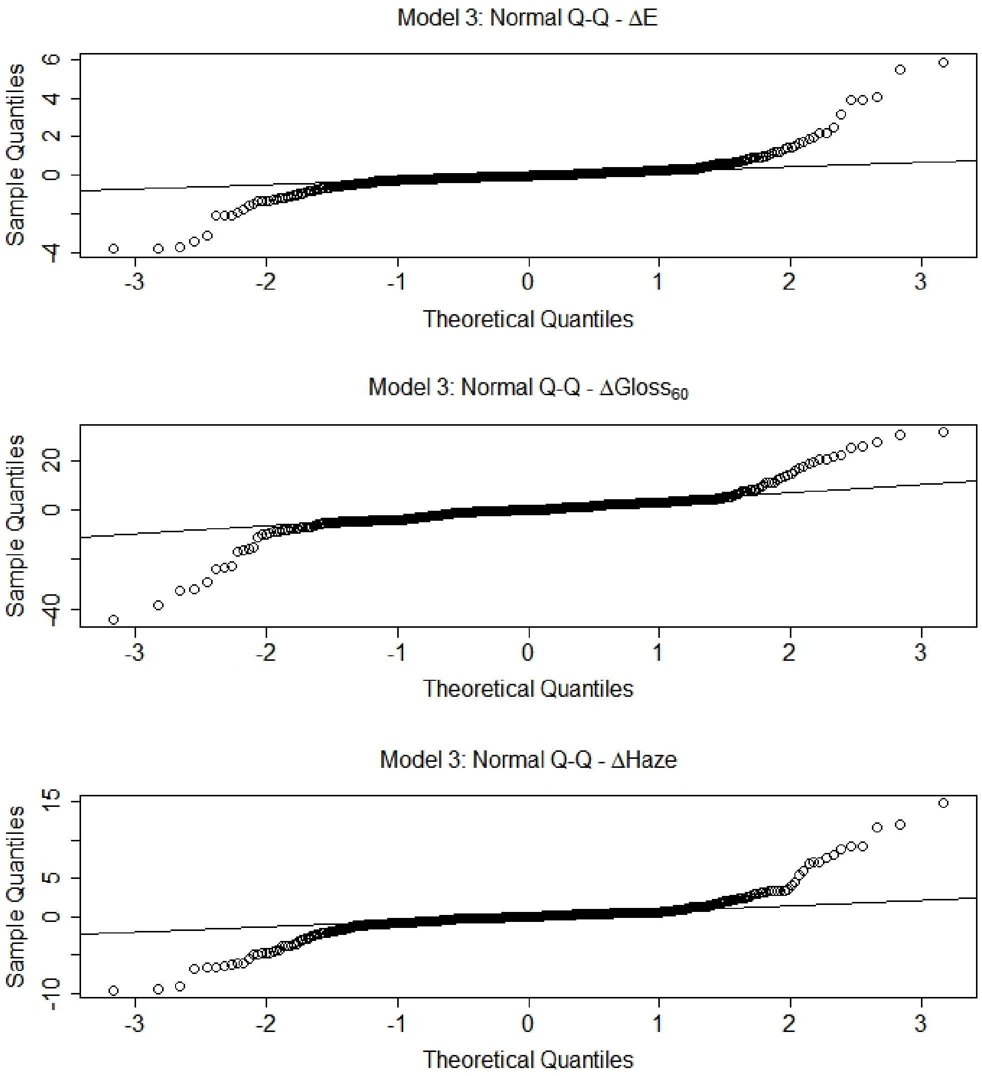

Fig D.2. Quantile-Quantile values diagnostic plots for ΔE, $\Delta Gloss_{60}$, and $\Delta Haze$ for Model C-Accelerated.

Appendix E

Table E.1. Coefficients for Model W-Accelerated. Y_1 and Y_2 represent ΔE and $\Delta Gloss_{60}$, respectively. q is the natural spline basis function counter for $UVA_{<360}$ photodose.

$q=0$	$\boldsymbol{\beta_{00}}$	$\boldsymbol{\beta_{01}}$	$\boldsymbol{\beta_{02}}$	$\boldsymbol{\beta_{03}}$	$\boldsymbol{\beta_{04}}$	$\boldsymbol{\beta_{05}}$	$\boldsymbol{\beta_{06}}$	$\boldsymbol{\beta_{07}}$
Y_1	0.06	0.14	0.13	0.05	0.04	-0.02	0.002	0.0002
Y_2	-0.01	-0.007	-0.04	0.07	0.07	-0.09	-0.01	0.0005
$q=1$	$\boldsymbol{\beta_{10}}$	$\boldsymbol{\beta_{11}}$	$\boldsymbol{\beta_{12}}$	$\boldsymbol{\beta_{13}}$	$\boldsymbol{\beta_{14}}$	$\boldsymbol{\beta_{15}}$	$\boldsymbol{\beta_{16}}$	$\boldsymbol{\beta_{17}}$
Y_1	-0.82	-1.42	2.25	1.34	0.81	-1.48	-0.68	0.07
Y_2	46.4	16.8	19.4	-7.18	1.90	27.7	-48.0	-1.26
$q=2$	$\boldsymbol{\beta_{20}}$	$\boldsymbol{\beta_{21}}$	$\boldsymbol{\beta_{22}}$	$\boldsymbol{\beta_{23}}$	$\boldsymbol{\beta_{24}}$	$\boldsymbol{\beta_{25}}$	$\boldsymbol{\beta_{26}}$	$\boldsymbol{\beta_{27}}$
Y_1	1.16	6.17	4.11	9.57	7.46	-4.00	-0.81	0.10
Y_2	-15.8	35.5	-7.23	6.03	11.8	21.0	-25.7	-0.50
$q=3$	$\boldsymbol{\beta_{30}}$	$\boldsymbol{\beta_{31}}$	$\boldsymbol{\beta_{32}}$	$\boldsymbol{\beta_{33}}$	$\boldsymbol{\beta_{34}}$	$\boldsymbol{\beta_{35}}$	$\boldsymbol{\beta_{36}}$	$\boldsymbol{\beta_{37}}$
Y_1	6.52	13.6	3.23	1.21	0.64	-0.61	-2.22	-0.046
Y_2	-66.1	54.9	-23.1	39.8	46.2	9.56	-15.2	-0.27

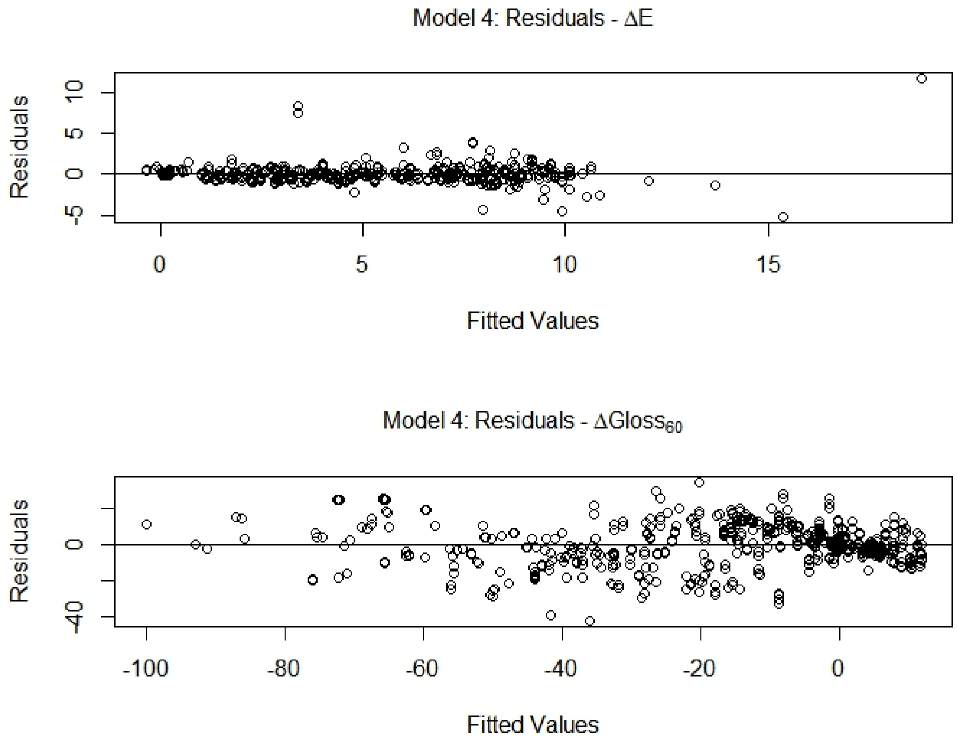

Fig E.1. Residuals versus fitted values diagnostic plots for ΔE and $\Delta Gloss_{60}$ for Model W-Accelerated.

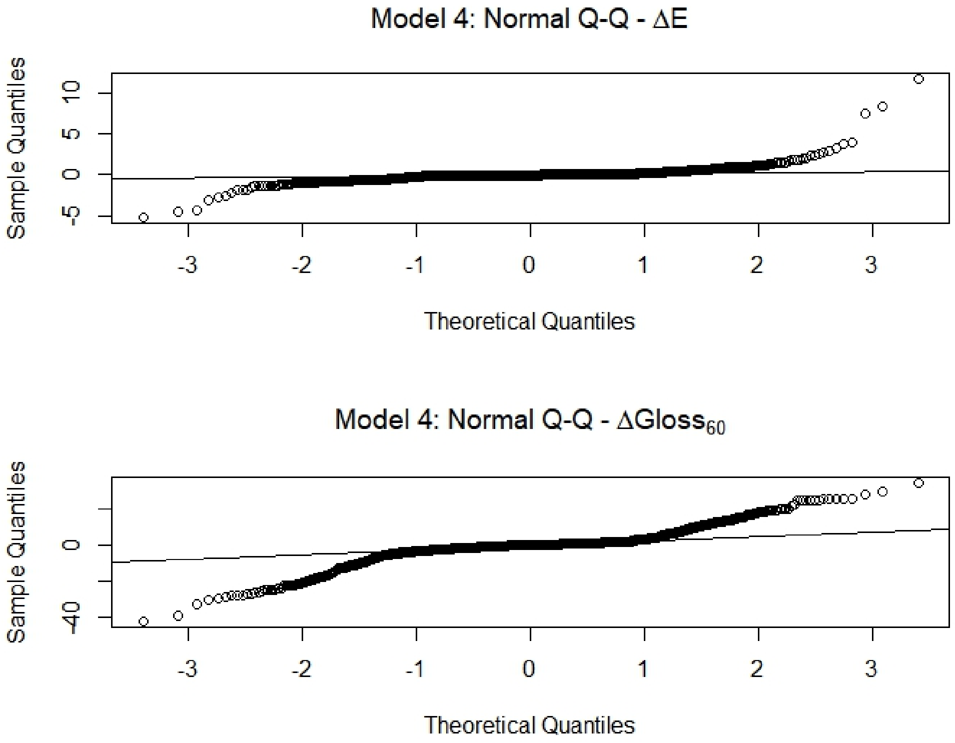

Fig E.2. Quantile-Quantile values diagnostic plots for ΔE and $\Delta Gloss_{60}$ for Model W-Accelerated.

Appendix F

MMR models were chosen over univariate regression models and generalized additive models (GAMs) because they balance the two necessities of goodness of fit and model interpretability. In this context, goodness of fit is defined as the ability of the model to predict or describe the underlying data. Model interpretability is the ability of the coefficients of the model terms to be understood in the context of the model's domain. Table F.1 shows the comparison of adjusted R^2 values from a simple univariate regression model without interaction terms, MMR, and generalized additive models fit to the outdoor clear PET degradation data.

Table F.1. Adjusted R^2 values for the three response variables for Model C-Outdoor, a simple regression model, and a GAM fit to the data for clear PET degradation under accelerated exposure.

	ΔE	$\Delta Gloss_{60}$	$\Delta Haze$
Simple	0.476	0.506	0.409
MMR	0.933	0.943	0.964
GAM	0.958	0.941	0.963

The adjusted R^2 values of the simple regression models were lower than those of the others. Simple models lack interaction terms; therefore, they don't capture the different degradation trends between materials very well, nor the synergistic effects between

stressors. For example, a simple model that includes a indicator variable to represent the presence of moisture contact and ignores interaction with UV photodose does not accurately represent the degradation process, because light is the primary source of stress and there is significant synergy between the two stressors. The simple model provides the highest level of interpretability because the effect of each term in the model is separate and can be assessed via the coefficients. In contrast, the GAMs showed high overall adjusted R^2 values. While GAMs fit the data well due to the combination of highly flexible spline bases and the lack of limitation on knot placement, they lack interpretability. GAM models do not provide any straightforward means of extracting coefficients for spline terms, so one cannot interpret the impact of each term. GAMs effectively function as supervised black-box models. MMR models balance fit and interpretability because good fits can be obtained, as shown by the relatively high adjusted R^2 values, and models are interpretable because coefficients are explicitly defined for univariate terms and interaction terms.

References

1. R Core Team. R: A Language and Environment for Statistical Computing; 2017. Available from: `https://www.R-project.org/`.

2. RStudio Team. RStudio: Integrated Development Environment for R; 2016. Available from: `http://www.rstudio.com/`.

3. Wickham H. ggplot2: Elegant Graphics for Data Analysis. Springer-Verlag New York; 2009. Available from: `http://ggplot2.org`.

4. Cheng R. qtlmt: Tools for Mapping Multiple Complex Traits; 2017. Available from: `https://CRAN.R-project.org/package=qtlmt`.

5. R Core Team. R: A Language and Environment for Statistical Computing; 2017. Available from: `https://www.R-project.org/`.

6. Fox J, Weisberg S. An R Companion to Applied Regression. 2nd ed. Thousand Oaks CA: Sage; 2011. Available from: `http://socserv.socsci.mcmaster.ca/jfox/Books/Companion`.

7. Venables WN, Ripley BD. Modern Applied Statistics with S. 4th ed. New York: Springer; 2002. Available from: `http://www.stats.ox.ac.uk/pub/MASS4`.

8. Wood SN. Fast stable restricted maximum likelihood and marginal likelihood estimation of semiparametric generalized linear models. Journal of the Royal Statistical Society (B). 2011;73(1):3–36.

9. Wood SN, N , Pya, S"afken B. Smoothing parameter and model selection for general smooth models (with discussion). Journal of the American Statistical Association. 2016;111:1548–1575.

10. Wood SN. Stable and efficient multiple smoothing parameter estimation for generalized additive models. Journal of the American Statistical Association. 2004;99(467):673–686.

11. Wood SN. Generalized Additive Models: An Introduction with R. 2nd ed. Chapman and Hall/CRC; 2017.

12. Wood SN. Thin-plate regression splines. Journal of the Royal Statistical Society (B). 2003;65(1):95–114.

13. from Jed Wing MKC, Weston S, Williams A, Keefer C, Engelhardt A, Cooper T, et al.. caret: Classification and Regression Training; 2017. Available from: https://CRAN.R-project.org/package=caret.

14. Bryant C, Wheeler NR, Rubel F, French RH. kgc: Koeppen-Geiger Climatic Zones; 2017. Available from: https://cran.r-project.org/web/packages/kgc/index.html.
